# Reduced glycoprotein hormone **β**5 links male aging and testosterone decline to increased adiposity

**DOI:** 10.1172/JCI192355

**Published:** 2026-02-03

**Authors:** Gengmiao Xiao, Aijun Qian, Zhuo Gao, Tingting Dai, Hui Liang, Shuai Wang, Mulan Deng, Yunjing Yan, Xindan Zhang, Xuedi Zhang, Yunping Mu, Jiqiu Wang, Aibo Gao, Huijie Zhang, Fanghong Li, Allan Zijian Zhao

**Affiliations:** 1 Department of Endocrinology and Metabolism, Nanfang Hospital, Southern Medical University, Guangzhou, China.; 2 Department of Endocrinology and Metabolism, Zhongshan Hospital, Fudan University, Shanghai, China.; 3 The School of Biomedical and Pharmaceutical Sciences, Guangdong University of Technology, Guangzhou, China.; 4 Department of Clinical Laboratory, the Fourth Affiliated Hospital of Harbin Medical University, Harbin, China.; 5 Hunan University of Medicine General Hospital, Huaihua, China.; 6 Department of General Surgery, The First Affiliated Hospital of Nanjing Medical University, Nanjing, China.; 7 Guangzhou Quality Supervision and Testing Institute, Collaborative Innovation Center for NQI-Quality Safety of Guangzhou, Guangzhou, China.; 8 Department of Endocrine and Metabolic Diseases, Shanghai Institute of Endocrine and Metabolic Diseases, Ruijin Hospital, Shanghai Jiao Tong University School of Medicine, Shanghai, China.; 9 The Institute of International Translational Medicine, GuangDong Engineering Technology Research Center of Metabolic Disorders Interdisciplinary Precision Prevention and Digital Healthcare, The Eighth Affiliated Hospital, Southern Medical University, Foshan, China.

**Keywords:** Aging, Endocrinology, Metabolism, Adipose tissue, Obesity, Sex hormones

## Abstract

Aging commonly causes decline of testosterone or estrogen, leading to overaccumulation of fatness in men and women, respectively. Although such a phenomenon can be readily explained by estrogen’s direct action on adipocytes in women, accumulative evidence does not support the direct action of testosterone in adipocyte lipid metabolism, suggesting there is a missing intermediary link. Herein, we propose that glycoprotein hormone β5 (GPHB5) is the intermediary linkage between testosterone and the regulation of adiposity. In clinical samples, blood levels of GPHB5 were correlated negatively with men’s ages and positively with circulating testosterone. Testosterone directly stimulated the expression of GPHB5 in cultured cells; pharmacological blockade of androgen receptor (AR) functions abrogated this effect. Knockout of AR led not only to development of obesity but also reduction of GPHB5 expression. Genetic ablation of GPHB5 in men, but not women, reduced the browning of white adipose tissue, diminished energy expenditure, and caused severe obesity. Importantly, elevated blood testosterone levels did not exert catabolic actions in GPHB5^–/–^ mice; yet, recombinant GPHB5 protein could stimulate energy expenditure and reduce adiposity. These results provide strong proof that GPHB5 is the “missing” intermediary hormone linking testosterone (and aging) and its well-known catabolic effect on adipose tissue.

## Abstract 2

Sexual dimorphic regulation of fat accumulation by glycoprotein hormone b5 during aging and testosterone decline.

## Introduction

It is a universally recognized phenomenon that, during aging, men start to gradually gain weight with central accumulation of adiposity as the prominent feature (1, 2). Today, this pattern of adiposity poses a serious threat to cardiovascular health and metabolic homeostasis (3). The aging process correlates with the decline of energy expenditure, as thermogenesis, reflected in body temperature, gradually decreases as men age (4). In fact, one must be following a consistent and rigorous exercise program to mitigate such accumulation of adiposity during aging. Even the same exercise discipline will not fend off fat accumulation in aging men as efficiently as in young men (5, 6). Among various postulated themes explaining this phenomenon during aging in men, the purported role of declining circulating testosterone levels has gained the most traction. Indeed, blood concentrations of testosterone in men decrease with age beyond 30 years (7–10). Castration leads not only to loss of some male features but also to drastic gain in adiposity (11). Accumulation of fat also frequently is observed in patients with prostate cancer who receive chemotherapies with drugs targeting androgen or androgen receptors (ARs) (12, 13). In a genetic model, complete blockade of testosterone functions through gene targeting of AR expression led to the phenotypes similar to those seen in castrated men, including the development of obesity (14, 15).

Despite these observations, testosterone is unlikely to play a direct role in energy balance and fat metabolism. There has been some controversy as to whether direct incubation with testosterone could stimulate lipolysis in adipocytes (16–18). Although some previous studies reported the inhibitory effect of testosterone on differentiation and lipid accumulation during prolonged incubation with 3T3-L1 pre-adipocytes (19), others have refuted this conclusion (20). Importantly, adipocyte-specific knockout of the AR gene did not cause mutant mice to gain additional weight or more adiposity than their WT littermates (21), suggesting it is unlikely that testosterone acts directly on adipocytes and that an intermediate player links circulating testosterone levels with adiposity and energy balance. Such a player is expected to be endocrine. Its circulating level should rise and ebb along with that of testosterone. Importantly, this putative factor should directly work on fat cells to control fat metabolism and even help “burn off” fat (vis-à-vis heat production) through browning of the white fat. In a bioinformatic scanning of adipocyte-specific knockout of various receptors, we noticed that the phenotypes of thyroid-stimulating hormone receptor (TSHR) mutation highly resemble those of AR^–/–^mutations (15, 22, 23). In addition to TSH, TSHR also has another high-affinity hormonal ligand, glycoprotein hormone subunit β5 (GPHB5), an evolutionarily conserved protein with unknown biological functions (24–26).

Herein, we propose that GPHB5 is the circulating hormone that serves as the physiological link between aging, circulating testosterone levels, and adiposity. GPHB5 and glycoprotein hormone subunit α2 (GPHA2) can dimerize to form a novel glycoprotein hormone, corticotroph-derived glycoprotein hormone (CGH), which is, interestingly, a 4-fold more potent ligand of TSHR than TSH (27). Both subunits are expressed in a variety of tissues, chief among which are brain, pituitary, and testis (24, 27, 28). GPHB5 alone can induce a TSHR-mediated cAMP response, but GPHA2 is unable to do so. The expression of GPHA2 is much higher than that of GPHB5 in humans and murine species, suggesting the regulation of CGH function mainly depends on the variation of GPHB5 production (26–28).

GPHB5 is highly conserved across different species (24, 25), which suggests it plays an important evolutionarily preserved function. In reported transgenic mouse models, overexpression of GPHB5 is resistant to the development of obesity induced by a high-fat diet, whereas overexpression of GPHA2 in mice results in no observable phenotypes related to body weight (27, 29). In this study, we combined some clinical studies with knockout animal models to specifically delineate the indispensable role of GPHB5 in linking aging, testosterone decline, energy balance, and accumulation of adiposity.

## Results

### The association of blood GPHB5 and testosterone in different age groups.

**Figure 1:**
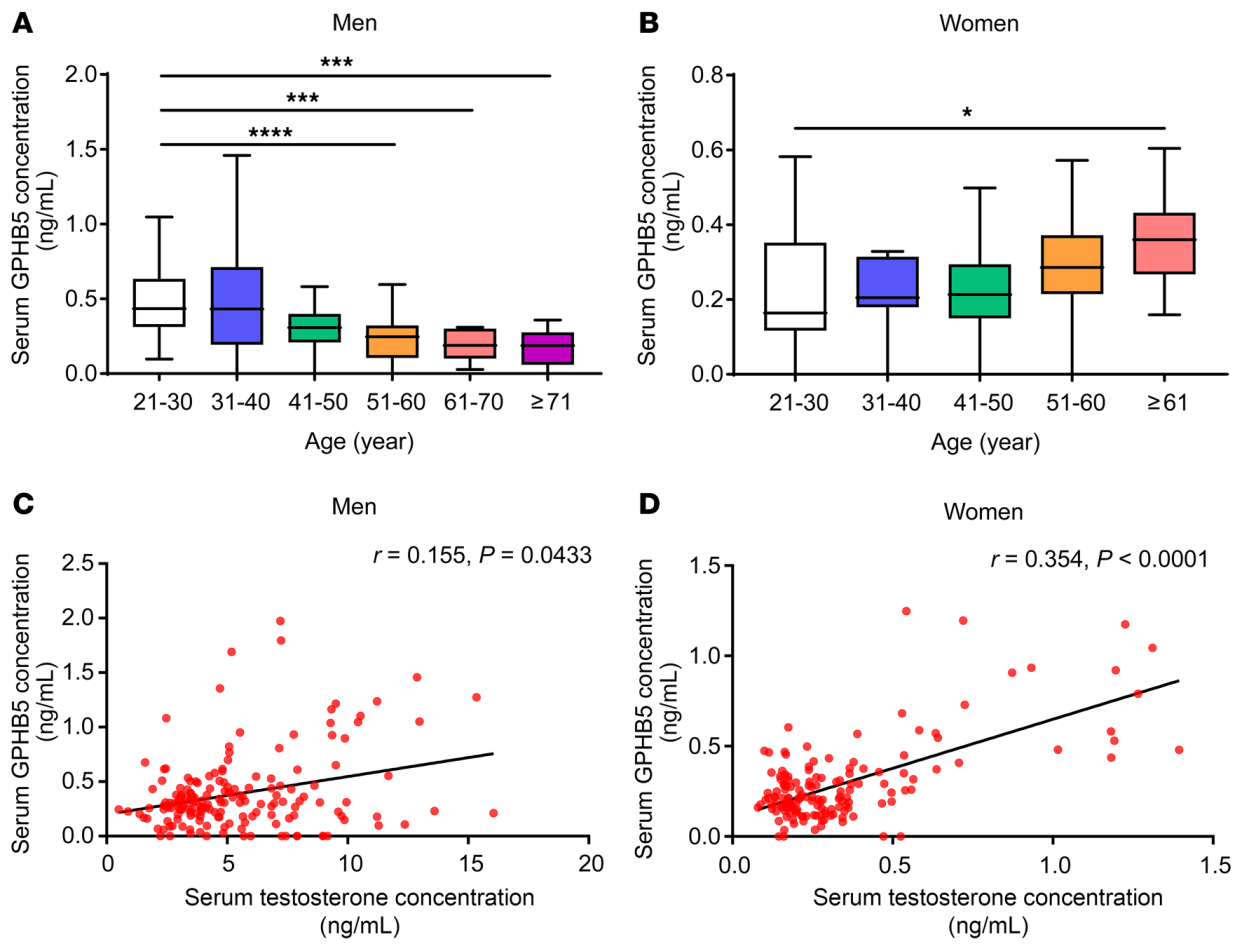
Serum GPHB5 levels in men and women during aging. Serum GPHB5 levels versus age in men (**A**) and women (**B**). The box plots display the 25th, 50th, and 75th percentiles (horizontal bars), with the whiskers extending to ± 1.5× IQRs. Correlation of testosterone with GPHB5 levels in men (**C**) and women (**D**). *n* = 171 for men and *n* = 152 for women. Statistical significance was determined by Kruskal-Wallis test with Dunn’s test for multiple comparisons (**A** and **B**); for correlation analyses, Spearman correlation coefficients (Spearman *r*) and *P* values are reported (**C** and **D**). *P < 0.05, ***P < 0.001, ****P < 0.0001.

As an initial attempt to evaluate the changes of GPHB5 in different age groups, we measured the blood levels of GPHB5 in samples collected from cohorts intended for regular physical checkup in 2 different local hospitals (see Supplemental Table 1; supplemental material available online with this article; https://doi.org/10.1172/JCI192355DS1). In men, circulating GPHB5 concentrations gradually decline with age, with a significant negative correlation (Figure 1A and Supplemental Figure 1A). In the blood samples, the median levels of GPHB5 reached 0.43 ng/mL in the young adult group (aged 21–30 years). In the age group between 51 and 60 years, the median value had declined to 0.25 ng/mL; this decline was even more drastic (to as low as 0.19 ng/mL) when we looked at the cohort of men of advanced age (older than 70 years) (Supplemental Table 1). In women, blood concentrations of GPHB5 were generally lower across different age groups (age 21–50 years) than those of the same age bracket until they reached advanced age (older than 60 years) (Supplemental Table 1). However, contrary to the findings in men, a significant positive correlation was identified with women’s ages (Figure 1B and Supplemental Figure 1B), primarily owing to the uptick in GPHB5 concentration in postmenopausal women. In the clinical cohort in this study, we did not find a significant correlation between GPHB5 concentration and fasting blood glucose, triglyceride (TG), total cholesterol (TC), low-density lipoprotein cholesterol (LDL-C), and high-density lipoprotein cholesterol (HDL-C) levels in men (Supplemental Figure 1, C–G) or women (Supplemental Figure 1, H–L).

Circulating testosterone concentrations decline with aging in men (7–9). Subsequent analysis of the total testosterone readings in the clinical samples from our cohort revealed a strong positive correlation with the blood concentrations of GPHB5 in men (Figure 1C), implying a potential regulatory role of testosterone on GPHB5 expression. In fact, a strong positive correlation also existed in women (Figure 1D). Curiously, serum GPHB5 was negatively correlated with circulating 17β-estradiol (Supplemental Figure 1M) in women. It appears that the circulating GPHB5 levels in women are the result of interplay between estrogen and testosterone. The data thus far prompted us to check the expression of GPHB5 in patients with polycystic ovary syndrome (PCOS), who usually have elevated testosterone concentrations (30). Using the published expression profiles in National Center for Biotechnology Information Gene Expression Omnibus (https://www.ncbi.nlm.nih.gov/geo/), we analyzed GPHB5 mRNA levels in the skeletal muscle (accession no. GSE8157) among patients with PCOS and healthy control participants without PCOS. The mRNA expression of GPHB5 in the samples of patients with PCOS was markedly higher than in those of the control group (Supplemental Figure 1N), further indicating a positive correlation between testosterone and GPHB5 in human blood samples.

### Testosterone regulates the production of GPHB5.

**Figure 2:**
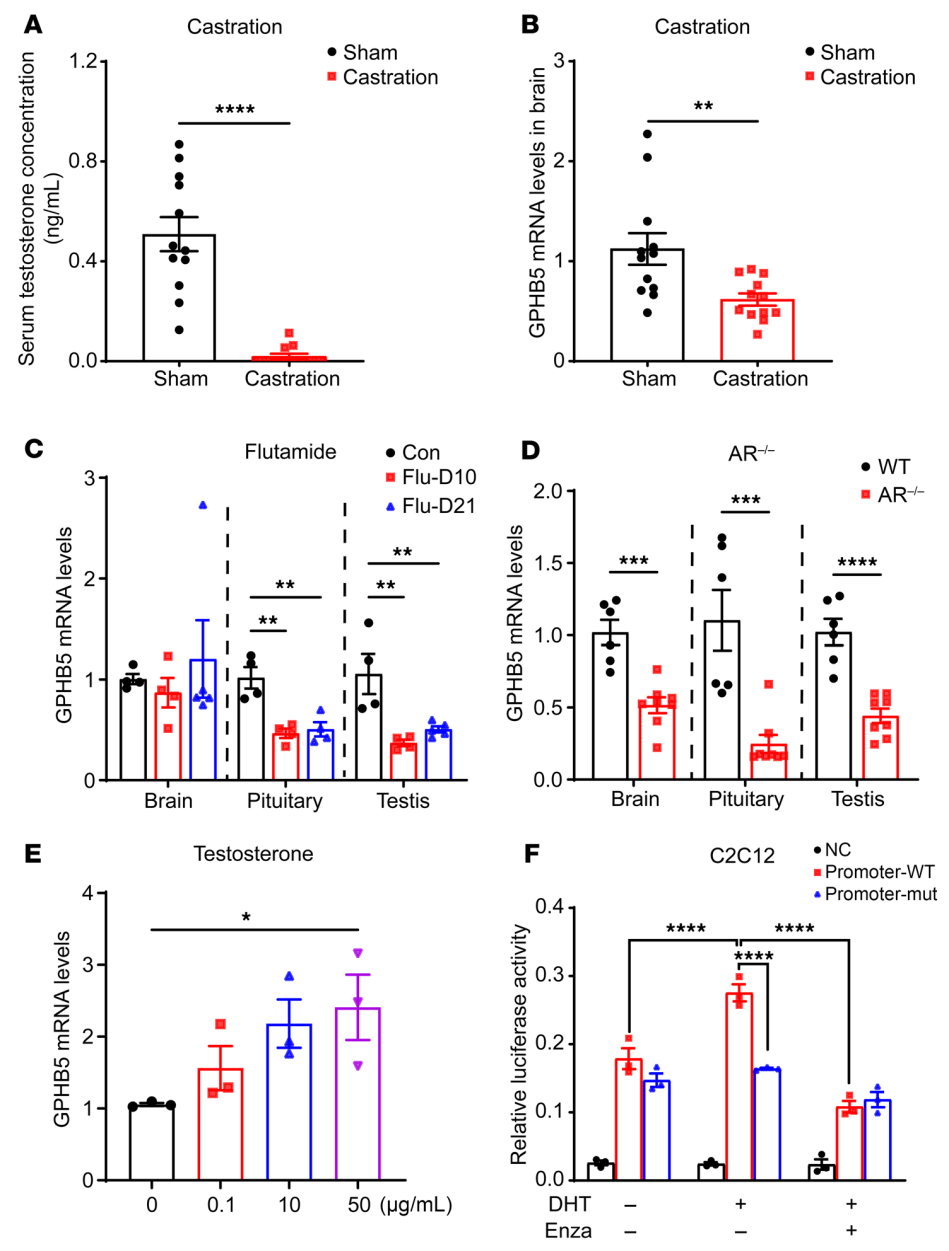
Testosterone regulated the production of GPHB5. The testosterone levels in serum (**A**) and GPHB5 mRNA levels in brain (**B**) of male mice (*n* = 12 per group). (**C**) GPHB5 mRNA levels in brain, pituitary, and testis of mice in which AR was inhibited by flutamide for 10 days and 21 days (*n* = 4–5 per group). Con, control. (**D**) GPHB5 mRNA levels in brain, pituitary, and testis of mice (*n* = 6 for WT and *n* = 8 for AR^–/–^ mice). (**E**) GPHB5 mRNA levels in C2C12 cells treated with testosterone for 1 day. (**F**) Relative luciferase activities in the C2C12 cells transfected with the WT and mutation GPHB5 promoter luciferase reporter plasmid. Cells were treated with DHT (10 nM) or enzalutamide (Enza; 0.1 μM) or both. NC, negative control. All data represent mean ± SEM; significant differences were determined by unpaired 2-tailed Student’s *t* test (**A**, **B**, and **D**); 1-way ANOVA with Dunnett’s multiple comparisons test (**C** and **E**); and 2-way ANOVA with Tukey’s multiple comparisons test (**F**). *P < 0.05, **P < 0.01, ***P < 0.001, ****P < 0.0001.

To verify that testosterone indeed directly regulates GPHB5 expression in vivo, we performed bilateral orchiectomy in male C57BL/6 mice to reduce blood testosterone concentrations (Figure 2A). Owing to the lack of a reliable GPHB5 ELISA kit to detect circulating GPHB5 in mouse blood samples, we examined the GPHB5 transcript levels in different tissues by real-time PCR. The castration led to a sharp drop in circulating testosterone levels and, importantly, a substantial decrease (~50%) of tissue GPHB5 expression in the same mice (Figure 2B), implying that testosterone is a crucial factor controlling GPHB5 expression; however, other regulatory factors are also involved.

To confirm the regulatory role of testosterone, we blocked the functions of AR through either the pharmacological inhibitor flutamide or gene deletion with CRISPR/Cas9 technology (AR^–/–^ mice) (Supplemental Figure 1O). Pharmacological blockade of AR caused marked diminution of GPHB5 expression in the tissue samples (e.g., pituitary, testis) (Figure 2C). Similarly, in the male AR^–/–^ mice, deletion of AR gene led to the reduction of GPHB5 expression (as evident in the brain, pituitary, and testis samples) (Figure 2D). These findings were corroborated by a cellular study in which co-incubation of testosterone with mouse myoblast C2C12 cells generated a dose-dependent stimulatory effect on GPHB5 expression (Figure 2E). However, in a parallel experiment, co-incubation of 17β-estradiol with the C2C12 cells did not alter GPHB5 expression (Supplemental Figure 1P), suggesting the regulation of GPHB5 is a sex-biased (or sex hormone–biased) physiological process.

To elucidate how testosterone exerts its direct stimulatory effect on GPHB5 expression, we systematically scanned the promoter region of human GPHB5 gene (https://jaspar.genereg.net/) for potential AR-binding sites and found 6 regions fitting as highly probable interaction sites (Supplemental Table 2). An expression plasmid with the luciferase reporter gene placed under the control of GPHB5 promoter was then transfected into the C2C12 cells. The luciferase reporter expression was greatly increased after addition of dihydrotestosterone (DHT), an AR agonist, in the transfected cells, but was dramatically tamed after incubation with the AR antagonist enzalutamide (Figure 2F). To further solidify the concept of direct regulation by AR, we mutated all the AR-binding sites of the GPHB5 promoter and found that neither DHT nor enzalutamide could affect GPHB5 expression (Figure 2F).

Although these findings cannot answer which AR-binding element is the most critical, they do demonstrate that testosterone directly stimulates the expression of GPHB5 via the AR-binding sites in the promoter of the GPHB5 gene. Taken together, these findings indicate the expression of GPHB5 is a result of direct regulation by testosterone, but not estrogen. Diminution of circulating testosterone levels, as in the case of male aging or blockade of ARs, will impede GPHB5 expression.

### GPHB5 deficiency leads to development of obesity.

We postulated that the decline of GPHB5 production is the primary cause of aging- and testosterone-waning–associated obesity in men and that GPHB5 serves as the intermediate player linking testosterone, energy balance, and adiposity. To test this hypothesis, we generated GPHB5-knockout (GPHB5^–/–^) mice with C57BL/6 background using CRISPR/Cas9 technology. Two independent lines of the knockout were generated, with both showing deletion of exons 2 and 3 (Supplemental Figure 2A), subsequently confirmed through a PCR analysis with specific primer sets (Supplemental Table 3). A systematic off-target analysis (combining bioinformatic predictions of the potential off-target sites and subsequent sequencing analysis) revealed no off-target deletion of genes (Supplemental Figure 2, B and C). The ablation of GPHB5 expression was also verified with a real-time PCR assay (Supplemental Figure 3A). Similar to the findings of previously published studies (31, 32), the knockout of GPHB5 did not influence the blood concentrations of either triiodothyronine (T3) or thyroxine (T4) when compared with those in the WT mice, even though GPHB5 or CGH uses TSHR as the canonical receptor (Supplemental Figure 3, B and C). We also confirmed that the deficiency of GPHB5 did not influence the expression of GPHA2 (the heterodimeric partner in CGH), further confirming the metabolic phenotypes were due to the mutation in GPHB5, not GPHA2 (Supplemental Figure 3D). Interestingly, the blood testosterone levels of the GPHB5^–/–^ mice were substantially elevated (Supplemental Figure 3E), implying a negative feedback loop of GPHB5 exists on testosterone production. Importantly, the elevated concentration of blood testosterone still could not exert its catabolic actions in the absence of GPHB5 expression (discussed later in this section).

**Figure 3:**
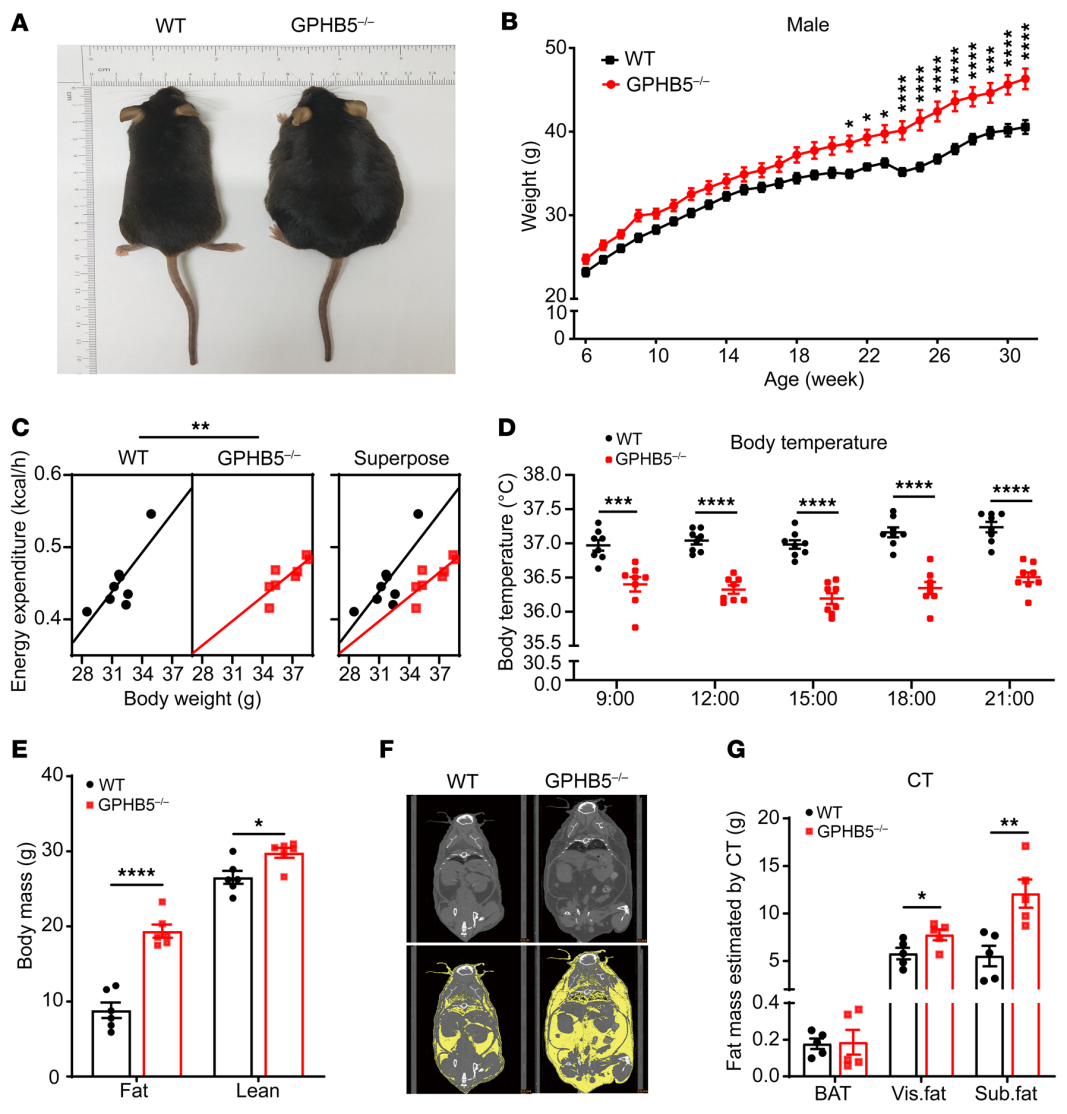
Lack of GPHB5 expression led to obesity in mice. (**A**) Pictures of male WT mice (left) and GPHB5^–/–^ mice (right) at 35 weeks of age. (**B**) Body weight curve for male mice (*n* = 18 for WT and *n* = 16 for GPHB5^–/–^ mice). The body weight of mice was tracked weekly from the age of 6 weeks to 31 weeks. (**C**) Energy expenditure in mice (*n* = 8 per group) was measured by indirect calorimetry and analyzed by ANCOVA. (**D**) Body temperature of mice (*n* = 8 per group) at room temperature (22°C). (**E**) Body fat mass and lean mass of mice were detected by EchoMRI (*n* = 6 per group). (**F**) Micro-CT images of WT mice (left) and GPHB5^–/–^ mice (right). The yellow parts in the coronal plane image represents WAT. (**G**) BAT between scapula, visceral fat (vis. fat), and subcutaneous fat (sub. fat) of mice (*n* = 5 per group) by micro-CT. All data represent mean ± SEM; significant differences were determined by 2-way ANOVA with Šídák’s multiple comparisons test (**B**); ANCOVA with energy expenditure as a dependent variable and body weight as covariate (**C**); and Student’s 2-tailed, unpaired *t* test (**D**, **E**, and **G**). *P < 0.05, **P < 0.01, ***P < 0.001, ****P < 0.0001.

Generation of the GPHB5^–/–^ mice allowed us to monitor the changes in body weight and adiposity over time. Compared with their WT littermates, the body weight of the male GPHB5^–/–^ mice diverged as early as 6 weeks of age, and these mice weighed more throughout the entire observation period (at least 31 weeks) (Figure 3, A and B). In contrast, very little difference in body weight was observed between the female GPHB5^–/–^ and their WT littermates (Supplemental Figure 3F), further confirming that the regulation of adiposity and body weight by GPHB5 is sexually dimorphic and that estrogen plays an expected prominent role in regulating female body weight.

Indirect calorimetry analysis showed that the energy expenditure of the male GPHB5^–/–^ mice was markedly lower than that of the WT control (Figure 3C and Supplemental Figure 3G), whereas respiratory exchange ratio (RER) and daily food intake were essentially the same between the 2 genotypes (Supplemental Figure 3, H and I). In this context, the reduced energy expenditure of the knockout mice was confirmed by ANCOVA, using body mass as a covariate, as previously reported (33, 34). Consistent with these observations, the body temperature of GPHB5^–/–^ mice was also markedly lower than that of WT mice (Figure 3D), further solidifying the notion that the increase in body weight of the male knockout mice was due to their reduced energy expenditure.

Using EchoMRI analysis to assess body composition, we discovered that the male GPHB5^–/–^ mice had sharply elevated fat mass and a small, yet substantial, increase in lean mass compared with the WT male littermates (Figure 3E). Such an increase in lean mass is likely attributed to the considerably elevated circulating testosterone levels in the knockout mice, because testosterone is well known to increase muscle mass and bone density (35, 36). By inference, the fat ratio in the overall body weight was also higher in the knockout mice than in the WT control mice (Supplemental Figure 3J), indicating that adipose accumulation was the primary cause of weight gain in the GPHB5^-/-^ mice. A whole-body micro-CT scan further verified that the body fat mass of the knockout male mice was considerably greater than that of the WT counterpart (Figure 3F and Supplemental Figure 3K), with both the visceral and subcutaneous fat accumulation as the prominent features (Figure 3G). Although the weights of ex vivo brown adipose tissue (BAT) dissected from the WT and GPHB5 mice were essentially the same, the weights of inguinal white adipose tissue (iWAT) and epididymal white adipose tissue (eWAT) of the knockout mice were much greater compared with those of their WT littermates (Supplemental Figure 3, L and M), a finding consistent with the results of the micro-CT scans.

### GPHB5 deficiency leads to reduced WAT browning and body temperature.

**Figure 4:**
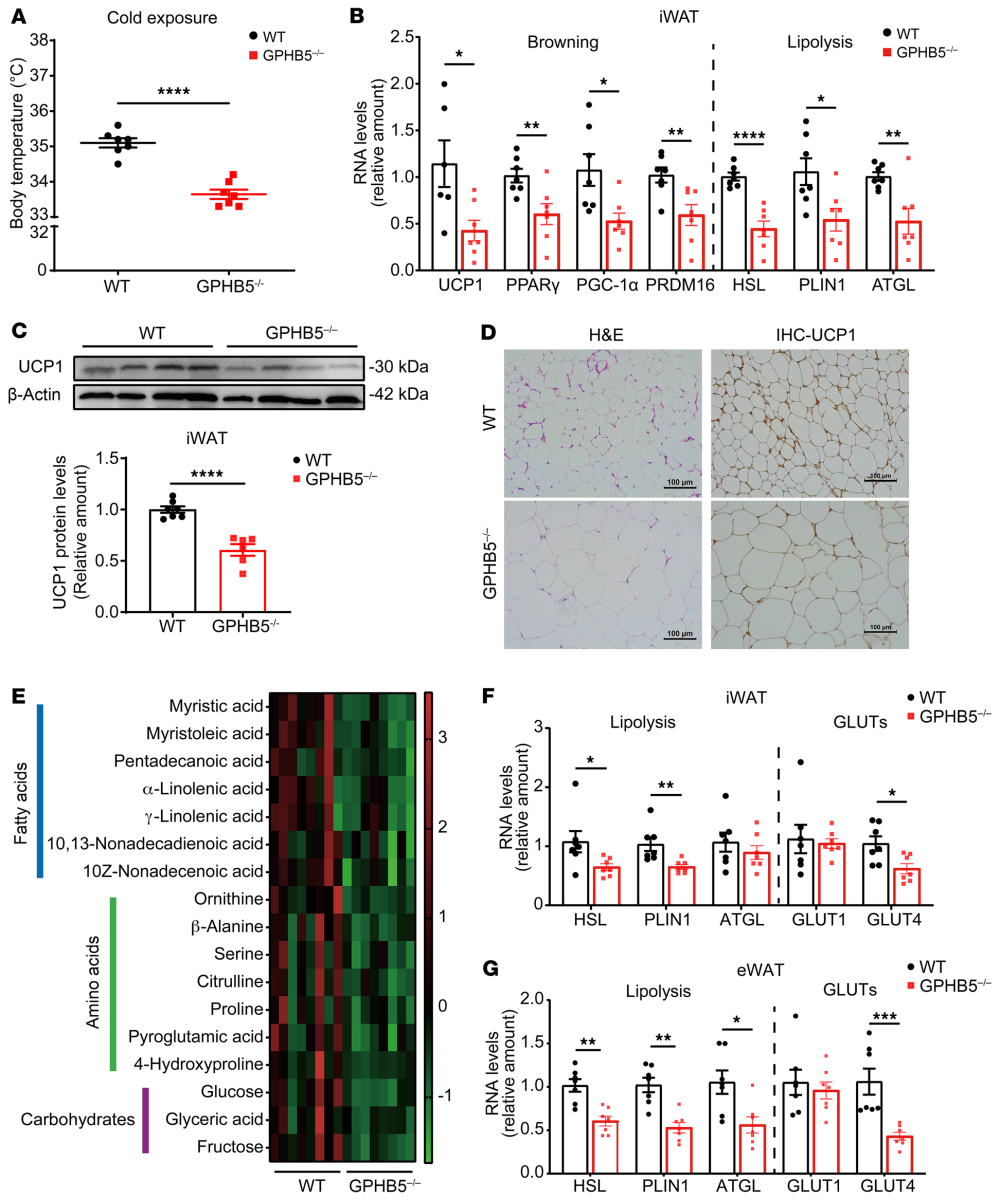
Mice lacking GPHB5 had decreased body temperature, browning, and fat metabolism. (**A**) Core body temperature of mice after cold exposure (4°C, 24 hours; *n* = 7 per group). (**B** and **C**) qPCR analysis of browning and lipolysis-associated genes, *n* = 6–7 per group (**B**) and Western blot analysis of UCP1, *n* = 7 for WT and *n* = 6 for GPHB5^–/–^ mice (**C**) in iWAT after cold exposure (4°C, 24 hours). Quantification of UCP1 protein levels also is shown. (**D**) Representative images of H&E-stained iWAT and UCP1-specific IHC of iWAT from mice after cold exposure (4°C, 24 hours; *n* = 7 per group). Scale bars: 100 μm. (**E**) Metabolite heatmaps of mice iWAT (*n* = 8 per group) at room temperature (22°C). Rows reflected normalized (*z*-score) metabolite concentrations. (**F** and **G**) qPCR analysis of lipolysis and GLUTs associated genes in iWAT (**F**) and eWAT (**G**) of mice (*n* = 7 per group) at room temperature (22°C). All data represent mean ± SEM; significant differences between treatments were determined by unpaired 2-tailed Student’s *t* test (**A** and **C**) or Mann-Whitney *U* test (**B**, **F**, and **G**). *P < 0.05, **P < 0.01, ***P < 0.001, ****P < 0.0001.

The decreased body temperature in GPHB5^–/–^ mice prompted us to investigate their thermogenic potential. After being exposed to a 4°C environment for 24 hours, both the knockout and WT mice had reduced body core temperature, but GPHB5 deficiency led to sharp drop in body temperature to 33°C–34°C (Figure 4A). Such a remarkable phenotype led us to speculate that GPHB5^-/-^ deficiency might have decreased white-fat browning. Indeed, after the cold exposure and dissection of subcutaneous iWAT, the expression of uncoupling protein 1 (UCP1), a prominent marker of browning, was markedly reduced at both the mRNA and protein levels (Figure 4, B and C). Consistent with such an observation, the mRNA expression levels of several important genes transcriptionally controlling the browning process, such as PPARγ, PPARγ coactivator 1-α, and PR-domain containing 16, were all markedly decreased (Figure 4B). In parallel assays, several lipolysis-promoting genes, such as hormone-sensitive lipase (HSL), perilipin 1 (PLIN1), and adipose triglyceride lipase, also exhibited lower expression in the iWAT relative to those in the WT tissues (Figure 4B). Histopathology assay and IHC both revealed considerably larger fat cells and tamed UCP1 staining in the samples from the GPHB5^–/–^ mice than those from the WT mice (Figure 4D). In contrast, we did not see the characteristics of BAT whitening, such as enlargement of lipid droplets or reduction of multilocular droplets, in the knockout mice. In addition, the expression of UCP1 and lipid metabolic genes was not altered in the BAT.

These findings indicated that GPHB5 knockout did not influence the thermogenic ability of BAT (Supplemental Figure 3, N and O). Thus, GPHB5 deficiency led to decreased thermogenic ability with attenuated fat browning and lipid degradation as the underlying mechanisms.

### GPHB5 deficiency leads to decreased fat metabolism.

Next, we further explored the metabolic changes within the adipose tissue of the GPHB5^-/-^ mice. To this end, we examined the changes of various metabolites in iWAT by mass spectrometry. Compared with the samples from the WT littermates, there were 24 metabolites with marked changes as a result of GPHB5 deficiency (Figure 4E and Supplemental Table 4), among which free fatty acid (FA) metabolite levels dropped sharply, supporting the finding that GPHB5 deficiency caused reduction of fat breakdown upon cold exposure (Figure 4B). Indeed, even when housed at room temperature (~22°C), the GPHB5^-/-^ mutation substantially dampened the expression of those genes inducing lipolysis in both the epididymal and subcutaneous WATs (Figure 4, F and G). In fact, the change of metabolites in the fat tissues of the knockout mice was reflected not just in lipids but also in amino acids and carbohydrates, among which the glucose content decreased surprisingly by 70% (Figure 4E and Supplemental Table 4). Consistent with this observation, the expression of glucose transporter type 4 (GLUT4) was also markedly reduced (Figure 4, F and G). Diminution of GLUT4 expression in the fat tissue not only contributed to the insulin-resistant phenotypes of the GPHB5-deficient mice (discussed next) but also reflected the preference of adipocytes toward lipid, not glucose, absorption.

### GPHB5 deficiency leads to strong insulin resistance and severe hepatic steatosis.

**Figure 5:**
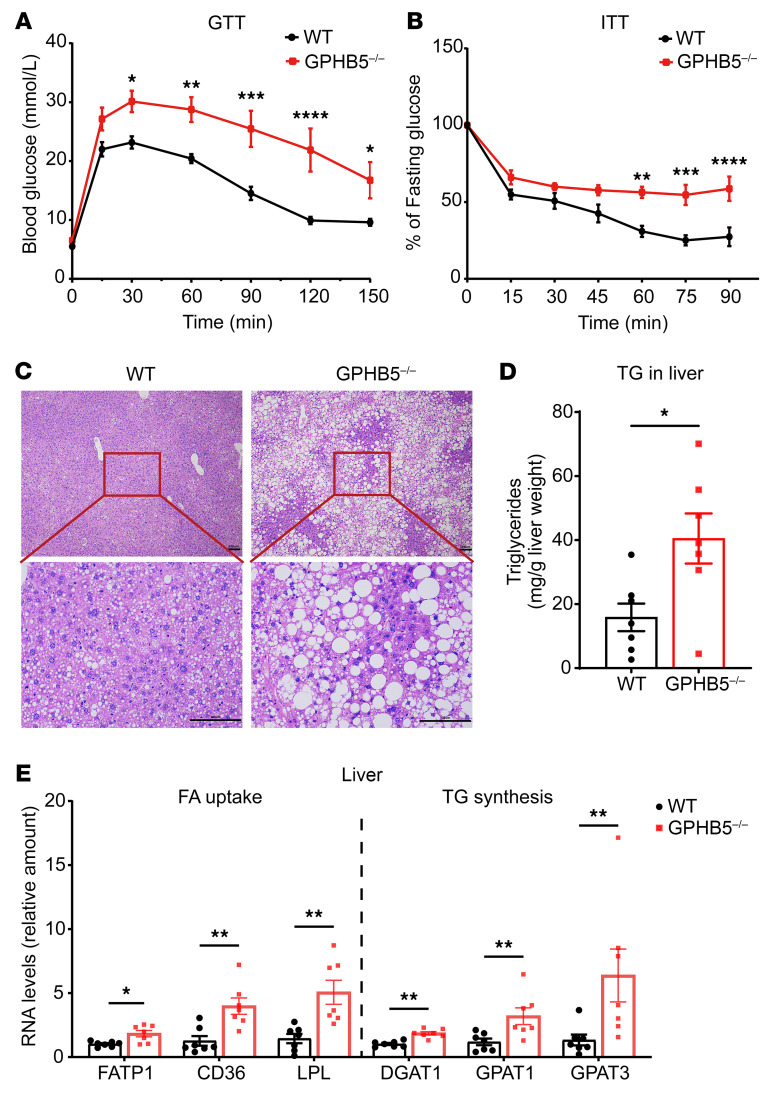
Lack of GPHB5 led to glucose intolerance, insulin resistance, and hepatic lipid accumulation. Glucose tolerance test (GTT) (**A**) and insulin tolerance test (ITT) results (**B**) of mice (*n* = 5 for WT and *n* = 4–5 for GPHB5^–/–^ mice). (**C**) Representative images of H&E-stained liver sections from WT and GPHB5^–/–^ mice. Scale bars: 100 μm. (**D**) TG levels in liver of mice (*n* = 7 per group). (**E**) qPCR analysis of FA uptake– and TG synthesis–associated genes in liver of mice (*n* = 7 per group). All data represent mean ± SEM; significant differences between treatments were determined by 2-way ANOVA with Šídák’s multiple comparisons test (**A** and **B**); unpaired 2-tailed Student’s *t* test (**D**); and Mann-Whitney *U* test (**E**). *P < 0.05, **P < 0.01, ***P < 0.001, ****P < 0.0001.

Even maintained with a regular chow diet, the GPHB5^–/–^ mice already displayed strong insulin resistance, as evident in the pronounced glucose intolerance in glucose tolerance tests (Figure 5A and Supplemental Figure 4A) and reduced insulin sensitivity in insulin tolerance tests (Figure 5B and Supplemental Figure 4B). GPHB5 deficiency also caused excessive ectopic fat deposition in the liver. H&E staining revealed severe steatosis and hepatocyte ballooning (Figure 5C) in the liver sections of GPHB5^–/–^ mice. The fatty liver phenotype was also quantitatively verified by the markedly increased hepatic TG content in the knockout mice (Figure 5D). Measurement of liver functions revealed that alanine aminotransferase levels were elevated and a trend toward increase in aspartate aminotransferase levels in the blood samples of GPHB5^–/–^ mice (Supplemental Figure 4, C and D), indicative of impaired liver functions.

Surprisingly, despite the heavy accumulation of fat, hepatic expression of those genes encoding the transporters for FA uptake, such as fatty acid transport protein 1, cluster of differentiation 36 (CD36), and lipoprotein lipase, were still markedly increased; the expression of some of genes increased sharply (Figure 5E). Furthermore, the expression of the enzymes for TG synthesis was also strongly elevated, such as for diacylglycerol *O*-acyltransferase 1 (DGAT1), glycerol-3-phosphate acyltransferase 1 (GPAT1), and GPAT3 (Figure 5E).

The strong insulin resistance phenotype further prompted us to evaluate the expression of those genes governing hepatic gluconeogenesis. Null mutation of GPHB5 substantially decreased hepatic expression of phosphoenolpyruvate carboxykinase and glucose-6-phosphatase, indicative of reduced ability of gluconeogenesis. In contrast, the hepatic expression of glucokinase, the enzyme playing critical roles in keeping glucose inside hepatocytes and subsequent glycogen synthesis, was substantially elevated (Supplemental Figure 4E). These changes, together, did not alter hepatic glycogen storage content in the GPHB5^–/–^ mice (Supplemental Figure 4F). Thus, GPHB5 deficiency caused not only development of obesity but also ectopic lipid deposition, as well as dysregulation of hepatic lipid synthesis and uptake.

### Reconstitution of GPHB5 leads to decreased adiposity in GPHB5^–/–^ or AR^–/–^ mice.

GPHB5 typically exists and functions through heterologous dimerization with GPHA2 (26, 27) to form CGH. To solidify the linkage between circulating GPHB5 level and testosterone, we first expressed recombinant human CGH protein (rCGH) in a Chinese hamster ovarian cell line and purified it to near homogeneity (Supplemental Figure 5A). In the initial test of the function of this recombinantly expressed product, differentiated 3T3-L1 adipocytes were incubated with 0 μM, 0.5 μM, and 1 μM rCGH for 24 hours. Oil Red O staining revealed that rCGH decreased the lipid content in mature adipocytes (Supplemental Figure 5, B and C). Following the treatment of rCGH, the reduction of TG content as well as the increase in glycerol release from adipocytes demonstrated the promotion of lipolysis by rCGH (Supplemental Figure 5, D and E). Immunoblotting further showed that the phosphorylation levels of lipolysis-promoting enzymes, such as HSL and PLIN1, were elevated in the differentiated 3T3-L1 adipocytes (Supplemental Figure 5, F and G). Collectively, these findings indicated CGH could directly control lipid accumulation in adipocytes by regulating lipolysis.

To further ascertain whether the metabolic phenotypes of GPHB5 were mediated through a TSHR-initiated cAMP/PKA pathway, we pretreated 3T3-L1 adipocytes with H89, a specific inhibitor of PKA. The rCGH-stimulated glycerol release from the adipocytes was completely inhibited by H89 (Supplemental Figure 5H) and blocked the inhibitory effect of rCGH on intracellular TG content (Supplemental Figure 5I), indicating that H89 was able to neutralize rCGH-induced lipid lipolysis. Although the phosphorylation levels of HSL, PLIN1, and cAMP response element binding protein (CREB) were markedly elevated after the treatment with rCGH, when co-incubated with H89, these effects of rCGH completely disappeared (Supplemental Figure 5, J–M). Taken together, these findings strongly supported a role of GPHB5 in lipid metabolism, which was dependent on cAMP/PKA signal transduction.

**Figure 6:**
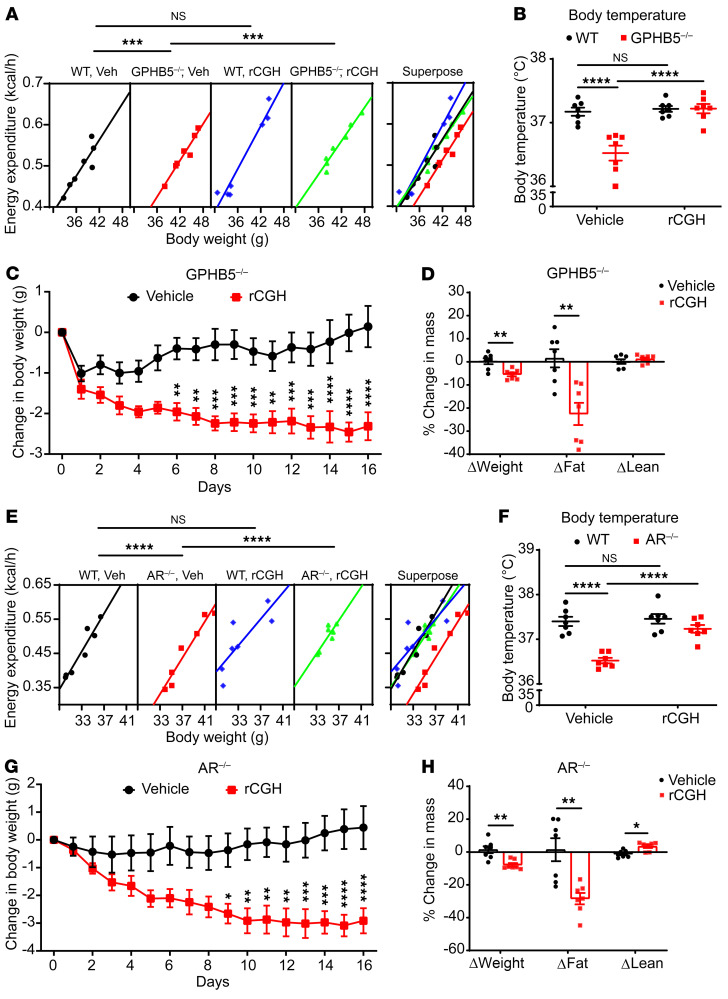
rCGH reversed the phenotypes of GPHB5^–/–^ and AR^–/–^ mice. Energy expenditure (**A**) and core body temperature (**B**) of mice treated with vehicle (Veh) or 10 mg/kg rCGH (*n* = 7 for WT and GPHB5^–/–^ mice). (**C**) Body weight change of GPHB5^–/–^ mice treated with vehicle or 5 mg/kg rCGH (*n* = 7 per group). (**D**) Percentage change in body weight, fat mass, and lean mass at day 16 compared with day 0 in GPHB5^–/–^ mice (*n* = 7 per group). (**E** and **F**) Energy expenditure (**E**) and core body temperature (**F**) of mice treated with vehicle or 10 mg/kg rCGH (*n* = 7 for WT and AR^–/–^ mice). (**G**) Body weight change of AR^–/–^ mice treated with vehicle or 5 mg/kg rCGH (*n* = 7 per group). (**H**) Percentage change in body weight, fat mass, and lean mass at day 16 compared with day 0 in AR^–/–^ mice (*n* = 7 per group). All data represent mean ± SEM; significant differences were determined by ANCOVA with energy expenditure as a dependent variable and body weight as covariate (**A** and **E**); unpaired 2-tailed Student’s *t* test (**D**); 2-way ANOVA with Šídák’s multiple comparisons test (**B**, **C**, **F**, and **G**); and Mann-Whitney *U* test (**H**). *P < 0.05, **P < 0.01, ***P < 0.001, ****P < 0.0001.

We initially evaluated the safety of rCGH treatment at the tested dose. In WT mice, intravenous delivery of rCGH (10 mg/kg) did not cause substantial changes in the blood biochemical indices, including liver and kidney functions (Supplemental Figure 6, A–F), indicating little toxicity. Reconstitution of rCGH in the GPHB5^–/–^ mice also did not influence the expression of GPHA2 (Supplemental Figure 6G), suggesting that any metabolic phenotype after rCGH treatment was unlikely due to the changes in GPHA2 activity. Next, male GPHB5^–/–^ mice and their WT littermates were intravenously given either vehicle control (PBS) or 10 mg/kg rCGH once and subjected to indirect calorimetry evaluation. Introduction of rCGH, but not the vehicle control, into GPHB5^–/–^ mice elevated energy expenditure (Figure 6A) and restored body temperature to a level equivalent to that of WT mice (Figure 6B). In contrast, the energy expenditure of WT mice did not change after the injection of rCGH (Figure 6A), further supporting that the observed effect of the rCGH was unlikely due to some nonspecific event.

We further investigated whether reconstitution of GPHB5 (in the form of CGH) can diminish adiposity, using a prolonged treatment module in the GPHB5^–/–^ mice. Consecutive daily intraperitoneal injection of rCGH or vehicle was performed for 16 days. At the end of treatment, GPHB5^–/–^ mice had a marked loss of total body weight by 5.5%, which was primarily due to a 22.6% decrease in fat mass (even with a modest increase in lean mass) (Figure 6, C and D). The diminishment of adiposity was primarily a result of increased energy expenditure and not the changes in RER and caloric intake (Figure 6A and Supplemental Figure 6, H and I). In parallel, the vehicle-treated group still had an increase in fat mass (Figure 6D).

While examining the expression of lipolysis-related genes in both iWAT and eWAT, we found that the prolonged treatment with rCGH markedly increased the phosphorylation levels of HSL and CREB in both iWAT and eWAT (Supplemental Figure 6, J–M) and, at least in iWAT, the treatment elevated the protein expression of HSL and PLIN1 and restored the expression of GLUT4 (Supplemental Figure 6, J and K). These results indicated that the prolonged daily delivery of the recombinant protein decreased adiposity by activating lipolysis in the adipose tissue.

A previous study revealed that blockade of testosterone function through gene deletion of AR led to reduced energy expenditure without changes in food intake (15). In the present study, we have also revealed the reduction of circulating GPHB5 in the AR^–/–^ mice. A crucial test is to see if restoration of GPHB5 (in the form of CGH) in the AR^–/–^ mice can alter energy metabolism, particularly by elevating energy expenditure. The AR^–/–^ and WT littermates were given, once, either the vehicle control or 10 mg/kg rCGH protein via intravenous injection. In the ensuing indirect calorimetry analysis, we found that the rCGH substantially increased energy expenditure in the AR^–/–^ mice but not in the WT mice (Figure 6E). The parallel vehicle treatment did not generate any ostensible change in energy expenditure in either the AR^–/–^ or WT mice (Figure 6E). Consistent with these findings, at the end of 24-hour observation in metabolic cages, the body temperature of the AR^–/–^ mice was elevated and nearly identical to that of the WT mice (Figure 6F).

To investigate the impact on adiposity following a prolonged treatment regimen with rCGH, AR^–/–^ mice were subjected to a consecutive daily treatment with rCGH for 16 days. Similar to the findings in GPHB5^–/–^ mice, the AR^–/–^ mice given rCGH had a marked reduction in both body weight and fat mass (Figure 6, G and H). CGH supplementation did not change food intake in the AR^–/–^ mice (Supplemental Figure 7A). Thus, introduction of rGPHB5 (in the form of CGH) increased energy expenditure despite the blockade of testosterone function.

We must emphasize that although both GPHB5 and CGH have been postulated to mimic the functions of TSH by stimulating the production of T3 and T4 in thyroid (26, 27), the treatment with rCGH in either GPHB5^–/–^ or AR^–/–^ mice did not result in an increase in serum T3, T4, and TSH concentrations (Supplemental Figure 7, B–G), demonstrating that the increase in energy expenditure and the reduction of fat mass was independent of thyroid hormones.

### GPHB5 regulates energy expenditure and adiposity in male ob/ob and female postmenopausal models.

**Figure 7:**
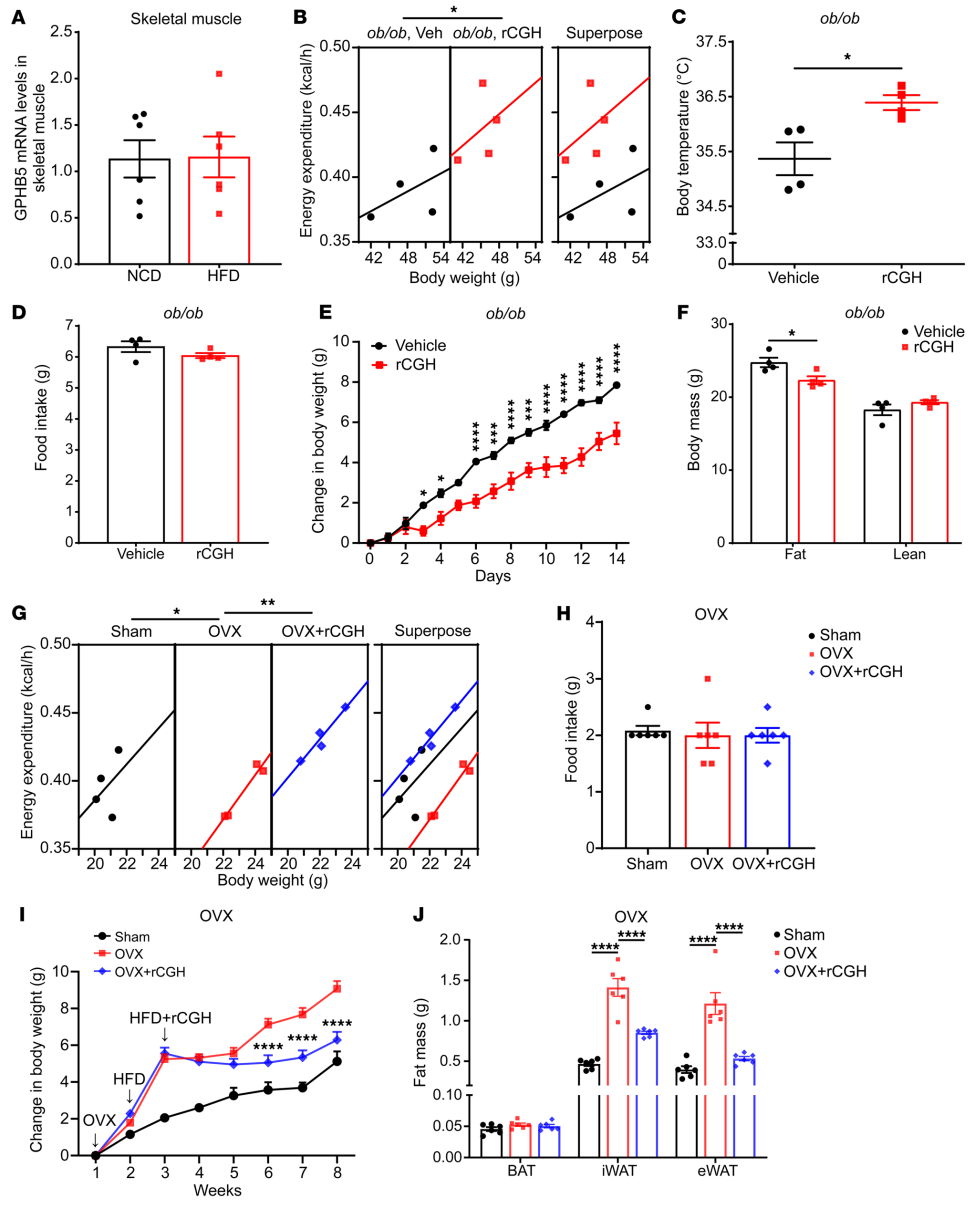
Impact of rCGH supplementation on metabolic parameters in *ob/ob* mice and OVX mice. (**A**) Quantification of GPHB5 expression in the skeletal muscle of mice with diet-induced obesity compared with control mice (*n* = 6 per group). (**B**–**E**) Energy expenditure (**B**), core body temperature (**C**), food intake (**D**), and body weight change (**E**) of *ob/ob* mice treated with vehicle (Veh) or 5 mg/kg rCGH (*n* = 4 per group). (**F**) Fat mass and lean mass of *ob/ob* mice treated with vehicle or 5 mg/kg rCGH (*n* = 4 per group). (**G**–**J**) Energy expenditure (**G**), food intake (**H**), body weight change (**I**), and fat mass (**J**) of OVX mice treated with vehicle or 5 mg/kg rCGH (*n* = 4–6 per group). All data represent mean ± SEM; significant differences were determined by unpaired 2-tailed Student’s *t* test (**A**, **C**, **D**, and **F**); ANCOVA with energy expenditure as a dependent variable and body weight as covariate (**B** and **G**); 1-way ANOVA with Dunnett’s multiple comparisons test (**H** and **J**); and 2-way ANOVA with Šídák’s multiple comparisons test (**E** and **I**). *P < 0.05, **P < 0.01, ***P < 0.001, ****P < 0.0001. HFD, high-fat diet.

We further investigated how GPHB5 expression might change in a general context of obesity, such as diet-induced obesity, in addition to androgen deficiency, such as the AR^–/–^ mouse model, and whether the physiological functions of GPHB5 can be expanded beyond GPHB5^–/–^ or AR^–/–^ models. Measurement of GPHB5 expression in the skeletal muscle tissues collected from mice fed a high-fat diet did not show marked alteration in GPHB5 expression (Figure 7A), indicating that obesity per se does not necessarily modify GPHB5 levels and that testosterone is still the inherently determining factor for GPHB5 production. On the contrary, administration of recombinant GPHB5 (in the form of rCGH) into *ob/ob* mice, a widely used model of severe obesity with genetic deficiency in leptin (37), generated similar metabolic phenotypes to those observed in GPHB5^–/–^ and AR^–/–^ mice. Accordingly, after rCGH supplementation in *ob/ob* mice, there was a marked increase in energy expenditure (Figure 7B), as evidenced by the elevated body temperature (Figure 7C). No alteration in food intake as a result of treatment was observed (Figure 7D). The elevation of energy expenditure led to a notable reduction in the rate of weight gain (Figure 7E). Importantly, rCGH-treated *ob/ob* mice had a reduction only in fat mass, without compromising lean mass (Figure 7F).

To further broaden the therapeutic potential of rCGH, particularly against obesity, in women, we tested its effects in an ovariectomy model (OVX) to simulate the metabolic dysfunctions and obesity in postmenopausal women. As expected, the energy expenditure of OVX mice substantially decreased compared with the sham-operated mice (Figure 7G). However, intravenous delivery of rCGH substantially increased the energy expenditure of OVX mice to the level of the sham group (Figure 7G). The quantity of food intake was not affected (Figure 7H), thus excluding the impact of energy-intake changes on the metabolic phenotype. Further analysis showed that rCGH treatment effectively restrained the growth of body weight in OVX mice (Figure 7I), demonstrating that rCGH can therapeutically suppress the body weight increase in the context of estrogen deficiency. rCGH did not change the weight of BAT, but markedly decreased the weight of iWAT and eWAT (Figure 7J), confirming that rCGH can reduce the quantity of WATs and attenuate the accumulation of body fat by promoting energy expenditure in the women with diminishing estrogen levels. Taken together, these findings indicate GPHB5 exerts a direct role in enhancing energy expenditure and reducing fat mass, which is not confined to the context of GPHB5^–/–^ or AR^–/–^ mice models, and that recombinant GPHB5 may be developed as a fat-reducing agent without affecting food intake.

## Discussion

Despite the long-held belief that the decline of testosterone underlies the gradual increase of adiposity across different mammalian species (13, 38–41), the accumulated experimental evidence does not support a direct regulatory role for testosterone on lipid metabolism. Although systemic null mutation of AR leads to development of obesity, adipocyte-specific deletion of the AR gene failed to generate an obese phenotype throughout the studied lifespan of animal models (14, 15, 21). Furthermore, the direct impact of co-incubation of testosterone on lipid metabolism in mature adipocytes has been disputed (16–18). Herein, we propose that GPHB5, a previously identified glycoprotein hormone, is the “missing” intermediary between the regulation of adiposity and the level of testosterone in men, particularly during aging or testosterone-decline.

Several lines of evidence presented here support the concept that testosterone stimulates the production of GPHB5. First, circulating GPHB5 levels negatively correlated with the ages of the studied population and, more importantly, had a positive correlation with blood testosterone level (even among women). Because it is difficult to follow the hormonal changes throughout a given individual’s life, such a significant correlation in our clinical cohort already provided strong evidence for our study. Second, at cellular level, testosterone can directly stimulate the expression of GPHB5 via some or all of the 6 AR-responsive elements in its promoter, mutations of which abrogate the stimulatory effect of testosterone. Third, impairment or deficiency of testosterone functions in the castrated or AR-knockout mice markedly decreased GPHB5 expression. Finally, acute inhibition of AR functions through pharmacological blockade also impaired GPHB5 expression both in vitro and in vivo, further supporting testosterone as a key regulator of GPHB5 homeostasis.

Functionally, co-incubation of rCGH with adipocytes led to stimulation of lipolysis. Genetic deletion of GPHB5-mutant male mice, but not female mice, did not result in changes in food intake, but did result in gross obesity, reduced fat browning and lipolysis, decreased energy expenditure and thermogenesis, severe insulin resistance, and hepatic steatosis. Interestingly, even though the knockout mice had elevated circulating testosterone levels, a feature typically associated with lean phenotype and reduced adiposity, they still developed severe obesity, which also strongly supports the concept that testosterone cannot stimulate fat metabolism without GPHB5 acting as a downstream mediator. In contrast, introduction of recombinant GPHB5 protein (as rCGH) into the AR-knockout or GPHB5^–/–^ mice elevated energy expenditure and reduced adiposity without loss of lean mass. Importantly, although the regulation of GPHB5 expression is dictated by testosterone, its catabolic effects are not necessarily limited to the context of GPHB5^–/–^ model or to the deficiency of testosterone function, such as the AR-deletion model. At least, in the *ob/ob* and OVX mice, rCGH resulted in restriction of body weight increase, elevated energy expenditure, and reduction in fat mass. The phenotypes were similar to those of the treated GPHB5- or AR-knockout models. These consistent observations across different models underscore a broad physiological role of GPHB5 in regulating energy balance and adiposity, and highlight its potential as a therapeutic modality for the treatment of obesity. Together, our data showed unequivocally that GPHB5 serves as an androgen-mediated intermediary hormone that controls adiposity.

Body weight, particularly adiposity, is regulated by a myriad of hormones, such as leptin, insulin, and GLP-1 (42). Herein, we propose that GPHB5 is also an important hormone that controls adiposity, but has a sex-biased pattern in the context of male aging and/or testosterone deficiency. The decrease in circulating GPHB5 level sends a strong signal for the body to entail multiple mechanisms to store fat, including slowing adipose browning, lipid turnover, and lipolysis, and some of these features are very similar to those in the AR^–/–^ situation and/or during aging in men (9, 43–46). However, the deficiency of GPHB5 led to a nearly “zealous” mobilization of energy source toward fat accumulation. In addition to severe obesity, there was also a sharp elevation of ectopic lipid buildup in the liver tissues even under regular diet. Despite such hepatic steatosis, the expression levels of FA transporters, in particular CD36 and FATP, as well as hepatic TG-synthetic enzymes (e.g., DGAT1, GPAT1/3), were still increased, which, in turn, would further propel hepatic accumulation of lipid. Evolutionarily, such a strong drive for fat storage is greatly beneficial to the survival of elderly men, so as to have enough energy store to survive the regular crisis of food shortage when their ability to hunt for food is diminished (47, 48). However, this survival virtue becomes a nondesirable trait in today’s “sheltered” environment, when abundant food supplies (49), coupled with decrease in energy expenditure induced by testosterone (and, therefore, GPHB5) decline, only leads to the development of obesity as well as obesity-associated metabolic syndromes in the context of aging.

Paradoxically, the physiological effect of GPHB5 on fat metabolism was sexually dimorphic. Perhaps it should not come as a surprise that women have lower blood GPHB5 concentrations than men, owing to their overall lower testosterone levels (50, 51). Consistent with the observations in humans, female GPHB5^–/–^ mice did not develop obesity, further demonstrating that GPHB5 is not a major hormone in female mice to regulate adiposity in aging. Systemic deficiency of both testosterone in men (13) and estrogen in women (52) leads to development of central obesity, yet only estrogen can directly inhibit adipogenesis and stimulate lipolysis by acting on its ERα receptor and subsequently inhibiting the activity of sterol regulatory element binding protein-1c as well as the expression of lipoprotein lipase in adipocytes (53, 54). In contrast to the lean phenotype in adipocyte-specific AR^–/–^ mice, the knockout of ERα in female mutant mice led to reduced lipolysis and obese phenotype, further solidifying the direct regulation of estrogen in adipocyte metabolism (55–57).

It is almost mystifying why testosterone, unlike its counterpart sex hormone, needs to elicit an intermediary player to regulate lipid metabolism in adipocytes. Testosterone can be easily converted to estrogen by aromatase (58) in adipocytes, which might be the reason authors of an earlier study observed stimulated lipolysis after extended incubation of testosterone with adipocytes (18), a condition that would also see dramatic buildup of estrogen in the medium (59). On the contrary, estrogen-to-androgen conversion was thermodynamically and enzymatically nearly impossible in mammals, although it was observed in a strain of the bacterium *Denitratisoma* sp., DHT3 (60), making it unlikely that estrogen can control GPHB5 expression via transforming into androgens. Unquestionably, the very existence of GPHB5 expression in females of species indicates this hormone may regulate other physiology processes as well. For example, GPHB5 can regulate ovarian reproductive function by increasing cAMP in ovarian granulosa cells in the presence of gonadotropins (61). The extent to which GPHB5 participates in the regulation of energy balance and other physiological actions in women remains to be elucidated. In addition, although circulating testosterone levels decline substantially in postmenopausal women (62), their circulating GPHB5 level is still elevated, suggesting testosterone is not the only primary factor controlling GPHB5 expression in women.

Such a sexual dimorphic regulatory pattern deserves further systematic investigation. Unfortunately, in our clinical cohort, a subset of clinical samples lacked full clinical parameters (~25% were missing complete glucose-lipid panels), making it difficult to quantitate the correlation between blood GPHB5 and other glucose and lipid metabolic readouts. However, such deficiency does not materially alter our primary conclusions that GPHB5 modulates adipose browning, lipolysis, and hepatic lipid, which are derived from robust, convergent in vivo and ex vivo experiments in our genetic and rescue models. Definitive clinical validation of the correlation analysis with a broad range of blood metabolic parameters will require much larger and full cohorts in future studies than the cohort we used.

Both GPHB5 and CGH (GPHB5/GPHA2 hybrid) and TSH can use TSHR as their canonical receptor for their respective biological functions (26, 27). In fact, CGH has greater affinity toward TSHR than TSH (26, 27). TSHR has been implicated in multiple metabolic processes in adipose tissues (22, 23, 63, 64). Activation of TSHR induced adipocyte lipolysis (63). Adipose-specific TSHR knockout resulted in obese phenotype and lower body temperature (22). Reintroduction of functional TSHR in the adipose tissue of TSHR-knockout mice restored lipolysis (23) and the expression of UCP1 (64). All these pathophysiological phenotypes are consistent with those in GPHB5^–/–^ or reconstitution models. Despite these observations, it is somewhat surprising to find that deletion of GPHB5 gene (reported here and in previous published studies;. refs. 31 and 32) had no impact on circulating levels of TSH, T3, and T4, nor did the infusion of CGH. Here, we offer a tentative structure-based explanation. The full-length TSHR (or the so-called uncleaved full length) can form a 2-subunit receptor (“cleaved”) entity after proteolytically cleaving off residues 311–377 (65, 66). Both the uncleaved and the cleaved form can be expressed in thyrocytes and nonthyroidal cells (66–69). Yet, upon binding to the cleaved TSHR, TSH can activate both cAMP and IP3 signaling pathways, which are necessary for thyroid hormone synthesis, whereas the uncleaved TSHR can only activate the cAMP signaling pathway (68). From a computational bioinformatic analysis (our unpublished data), we found that the free energy of CGH binding toward uncleaved TSHR is more than 4 times that of the binding to the cleaved form. Hence, we postulate that, thermodynamically, CGH prefers to bind to the uncleaved TSHR, which, in turn, causes the failure of CGH to stimulate the synthesis of thyroid hormones, owing to its inability of activating IP3 signaling pathway. Further experimental investigations are needed to test this theory.

In summary, GPHB5 is the crucial intermediary hormone that mediates the impact of testosterone on lipid metabolism and adiposity. Supplementation with rCGH can normalize body weight and reduce adiposity (but not lean mass) by stimulating energy expenditure without affecting thyroid function or energy intake in the context of testosterone decline and male aging. Thus, our study revealed a potential pharmaceutical agent for delaying the harmful sequelae of fat accumulation and decreased body temperature during aging, especially in today’s obesity-pandemic environment (3). Given that TSHR is expressed in a wide range of tissues, including the pancreas, testis, heart, and brain (70–73), additional profound physiological functions of GPHB5 should be explored.

## Methods

### Sex as a biological variable.

We examined male and female animals in this study, and sex-dimorphic effects are reported.

### Collection of human serum.

The study was approved by the Ethics Committee of the Fourth Affiliated Hospital of Harbin Medical University (ethics approval 2020-SCILLSC-09) and registered with the Chinese Clinical Trial Registry (https://www.chictr.org) as ChiCTR2000039136. We conducted this clinical research in accordance with Declaration of Helsinki principles. Human serum was obtained from the Fourth Affiliated Hospital of Harbin Medical University and Ruijin Hospital (Shanghai Jiao Tong University School of Medicine) after participants gave informed consent. All blood samples were drawn from the cubital vein of volunteers and allowed to coagulate for 30 minutes at room temperature before being centrifuged for 5 minutes at at ~1,509*g* (3,000 rpm). The serum was then aliquoted into tubes and frozen at –80°C until analysis.

### Animal models.

The GPHB5 and AR-knockout mice (C57BL/6N background) were generated by Cyagen Biosciences using CRISPR/Cas9 technology and housed in a specific pathogen–free environment. The body weights of GPHB5^–/–^ mice and WT littermates were tracked weekly. Male *ob/ob* mice were purchased from GemPharmatech Co. For the diet-induced obese model, 8-week-old C57BL/6 male mice were fed a high-fat diet for 12 weeks to induce obesity. The castrated mice (including male [by bilateral orchiectomy] and female mice), with the sham operation performed in a separate group. For the mouse model of AR inhibition, we used 5 mg/kg flutamide (Sigma, F9397) by intragastric gavage once daily for 10 and 21 days. Mice were sacrificed by cervical dislocation.

### Analysis of the off-target effect.

We designed PCR primers for the 10 most likely predicted off-target sites (Supplemental Table 3). The PCR products were sequenced and compared with the National Center for Biotechnology Information database; the results indicated no off-target disruption of non-GPHB5 sequences.

### Cell culture.

Mouse myoblast C2C12 cells (catalog SCSP-505) and 3T3-L1 preadipocytes (catalog SCSP-5038) were purchased from the Cell Bank of the Chinese Academy of Sciences in Shanghai. Cells were maintained at 37°C with 5% CO_2_ in DMEM (Gibco, 12800017) supplemented with 10% FBS (Gibco, 10091148) or 10% newborn calf serum (Bioind, 04-102-1A), and 1% penicillin–streptomycin (Gibco, 15140122). C2C12 cells were treated with 0 ng/mL, 100 ng/mL, 10 μg/mL, and 50 μg/mL testosterone (Wako, 207-20553), or 0 ng/mL, 0.1 ng/mL, 10 ng/mL, and 1,000 ng/mL 17β-estradiol (Sigma, E2758-1G) for 24 hours before collection for further analysis of gene expression. For differentiation, 3T3-L1 preadipocytes were cultured as described (74). The differentiated 3T3-L1 adipocytes were then co-incubated with 0 μM (PBS), 0.5 μM, and 1 μM rCGH for 24 hours. Then the TG content and glycerol release were detected by the Triglyceride Quantification Kit (Sigma, MAK266) and the Adipolysis Assay Kit (Cayman, 10009381), respectively.

### Oil Red O staining.

Differentiated 3T3-L1 cells were fixed with 4% paraformaldehyde (Servicebio, G1101) and stained in 60% original Oil Red O (Solarbio, G1260) for 15 minutes. Lipid content was quantified by detecting the OD values at 450 nm after solubilizing the stained cells with isopropanol (Aladdin, I112014).

### Luciferase reporter gene assay.

The human GPHB5 promoter was inserted into the firefly luciferase reporter plasmid GV238 (GeneChem), designated as promoter-WT. The AR-binding sites in GPHB5 promoter regions were predicted by the JASPAR database and mutated to be cloned into GV238 vector, which was named promoter-mut. C2C12 cells were cotransfected with 0.3 μg promoter-WT or protomer-mut plasmid, human AR overexpression plasmid, and *Renilla* luciferase plasmid CV045 (GeneChem) for 12 hours by Lipofectamine 8000 (Beyotime, C0533). After transfection, cells were treated with or without 10 nM DHT (Selleck, S4757) and 100 μM enzalutamide (Selleck, MDV3100) for 24 hours. Firefly and *Renilla* luciferase activities were measured with the Dual-Lumi II Firefly Luciferase Reporter Gene Detection Kit (Beyotime, RG089S), and the firefly luciferase activity was normalized to the *Renilla* luciferase activity.

### Measurements of body composition and temperature.

The fat mass and lean mass of the mice were determined by the EchoMRI-100 instrument (Echo Medical Systems), and the contents of BAT between the scapula, visceral, and subcutaneous fat were detected by micro-CT (Aloka, LCT-200). Body temperature was measured by an anal thermometer (JiNuoTai).

### Indirect calorimetry assay.

Energy expenditure, oxygen consumption, CO_2_ production, and RER were quantified by the indirect calorimetry system (Sable, PRO-MRMR-4).

### Cold-exposure experiment.

The mice were housed in a temperature-controlled environment at 12°C and maintained for 2 weeks for cold adaptation before being transferred to 4°C for 24 hours.

### Histology analysis.

Subcutaneous WAT was fixed in 4% paraformaldehyde (Servicebio, G1101), paraffin-embedded, and sectioned (5 μm) prior to H&E staining or IHC staining with an antibody against UCP1 (Cell Signaling Technology, 14670S). The liver and BAT were sectioned (3 μm) prior to H&E staining.

### Western blot analysis.

Adipose tissue and 3T3-L1 cells were lysed with RIPA buffer (Beyotime, P0013B) containing 0.1 M PMSF (Beyotime, ST505). PageRuler Prestained Protein Ladder (Thermo Fisher Scientific, 26616) was used as size marker. Total protein extract was separated by SDS-PAGE and transferred to PVDF transfer membranes. The membranes were probed with antibodies against UCP1 (Cell Signaling Technology, 14670S), HSL (Cell Signaling Technology, 18381S), phospho-HSL (Cell Signaling Technology, 4139S), PLIN1 (Cell Signaling Technology, 9349S), phospho-PLIN1 (Vala Sciences, 4856), GLUT4 (Cell Signaling Technology, 2213S), CREB (Cell Signaling Technology, 9197S), phospho-CREB (Cell Signaling Technology, 9198S), GAPDH (Cell Signaling Technology, 2118S), or β-actin (Sigma, A5316). Reactive bands were visualized by ECL Western Blotting Substrate (Thermo Fisher Scientific, 32106) and quantified by ImageJ software (NIH).

### RNA extraction and real-time PCR analysis.

Total RNA was extracted from tissues by Trizol (Sigma-Aldrich, T9424), and cDNA was synthesized with the HiScript III RT SuperMix (Vazyme, R323-01) according to the manufacturer’s instructions. For quantitative SYBR Green PCR assays, the ChamQ SYBR qPCR Master Mix (Vazyme, Q311-03) or Hieff qPCR SYBR Green Master Mix (Yeasen, 11201ES08) were applied in the assay reaction mix. Relative mRNA expression was calculated by the comparative Ct method and normalized to β-actin or TATA box-binding protein values measured in each sample.

### Glucose and insulin tolerance tests.

For glucose tolerance tests, the mice were fasted overnight and injected intraperitoneally with 2 g/kg 20% glucose. Blood glucose levels were measured by a glucometer (Accu-Chek Performa) at 0, 15, 30, 60, 90, 120, and 150 minutes after injection. For insulin tolerance tests, the mice were fasted for 6 hours and intraperitoneally injected with 0.8 IU/kg insulin (Novolin). Blood glucose levels were measured at 0, 15, 30, 45, 60, 75, and 90 minutes after injection.

### Measurement of serum parameters and TGs in liver.

In human participants, serum GPHB5 levels were measured with the human GPHB5 ELISA kit (MyBioSource, MBS280386), and serum total testosterone levels were measured with the Testosterone Parameter Assay Kit (R&D Systems, SKGE010) or Testosterone High-Sensitivity ELISA Kit (Enzo, ADI-900-176), following manufacturers’ protocols. For mouse samples, the levels of serum total testosterone, T3, and T4 were quantified using enzyme immunoassay kits, including the Testosterone Parameter Assay Kit (R&D Systems, SKGE010), T3 (Mouse/Rat) ELISA Kit (Bio Vision, K7422-100), and T4 (Mouse/Rat) ELISA Kit (Bio Vision, K7421-100). Alanine aminotransferase and aspartate aminotransferase levels were measured using the Automatic Biochemistry Analyzer (Chemray 800). TG content in liver was measured with the Triglyceride Quantification Kit (Sigma, MAK266).

### Metabolite measurement.

iWAT from WT and GPHB5^–/–^ mice was used for metabolomics analysis with a Q300 kit (Metabo-Profile). Profiling of all targeted metabolites was performed on an ultraperformance liquid chromatography-tandem mass spectroscopy system (ACQUITY UPLC-Xevo TQ-S, Waters Corp.) by Metabo-Profile Biotechnology, as previously reported (75).

### Recombinant human GPHA2/GPHB5 protein.

ExpiCHO-S Cells (Thermo Fisher Scientific, A29127) were transfected with expression constructs containing human GPHA2 and GPHB5 with CTP linker and His-tag. The recombinant GPHA2/GPHB5 (CGH) protein was purified from medium using AKTA pure Protein Purification System (GE Healthcare, now Cytiva). For desalting, we used a 3 kDa ultrafiltration tube (MilliporeSigma, UFC900396) and washed with PBS (2 mM KH_2_PO_4_, 10 mM Na_2_HPO_4_, 2.7 mM KCl, 137 mM NaCl) 3 times.

### Statistics.

Statistical analysis was evaluated by GraphPad Prism, version 8.4.3, or SPSS, version 26 (IBM). Non-normally distributed clinical data are presented as median (IQR), and statistical significance was assessed with the Kruskal-Wallis test. Other results were expressed as mean ± SEM. When comparing 2 groups, data were confirmed for normality and homogeneity of variance before using unpaired 2-tailed Student’s *t* test, otherwise the Mann-Whitney *U* test was used. One-way ANOVA and 2-way ANOVA were used when comparing 3 or more groups. Energy expenditure data were compared by ANCOVA, using body weight as a covariate.

### Study approval.

Animal experiments were approved by the Experimental Animal Ethics Committee of Guangdong Pharmaceutical University at Guangzhou (approval SPF2017038). Human studies were approved by the Ethics Committee of the Fourth Affiliated Hospital of Harbin Medical University (Ethics approval 2020-SCILLSC-09). All human participants provided written informed consent.

### Data availability.

The RNA-Seq data relating to Supplemental Figure 1N used the publicly available published dataset GSE8157 (76). Source data are provided in the Supporting Data Values file.

## Author contributions

AZZ and FL designed the study, supervised all aspects of the study, and contributed to writing the manuscript. GMX conducted experiments and acquired and analyzed the data. AJQ assisted with the expression, purification, and test of recombinant human CGH protein. ZG, TTD, HL, JQW, and ABG collected human serum samples. SW, MLD, YJY, Xindan Zhang, and Xuedi Zhang assisted with animal experiments. HJZ and YPM helped improve this manuscript during the revision process.

## Funding support

- The Noncommunicable Chronic Diseases-National Science and Technology Major Project (grant 2023ZD0506904 to AZZ).
- The Science and Technology Program of Guangzhou (grant 2023B03J1291 to AZZ).
- The Key Research and Development Program of Guangdong Province for “Innovative drug creation” (grant 2019B020201015 to FL).
- National Natural Science Foundation of China (grant 82100064 to YM).
- Key Research and Development Program of Guangdong Province (grant 2019B020227003 to FL).
- Scientific Research Start Plan of the Eighth Affiliated Hospital, Southern Medical University (grant SRSP2025RC01 to AZZ).

## Supplementary Material

Supplemental data

Unedited blot and gel images

Supporting data values

## Version 1 02/03/2026

In-Press Preview

## Version 2 03/16/2026

Electronic publication
