# Supplementary material for: Reduced glycoprotein hormone β5 links male aging and testosterone decline to increased adiposity
Source: J Clin Invest. 2026 Feb 3;136(6):e192355. doi: 10.1172/JCI192355 (PMC12987616; doi:10.1172/JCI192355)
Supplement: Supplemental data [file jci-136-192355-s127.pdf]

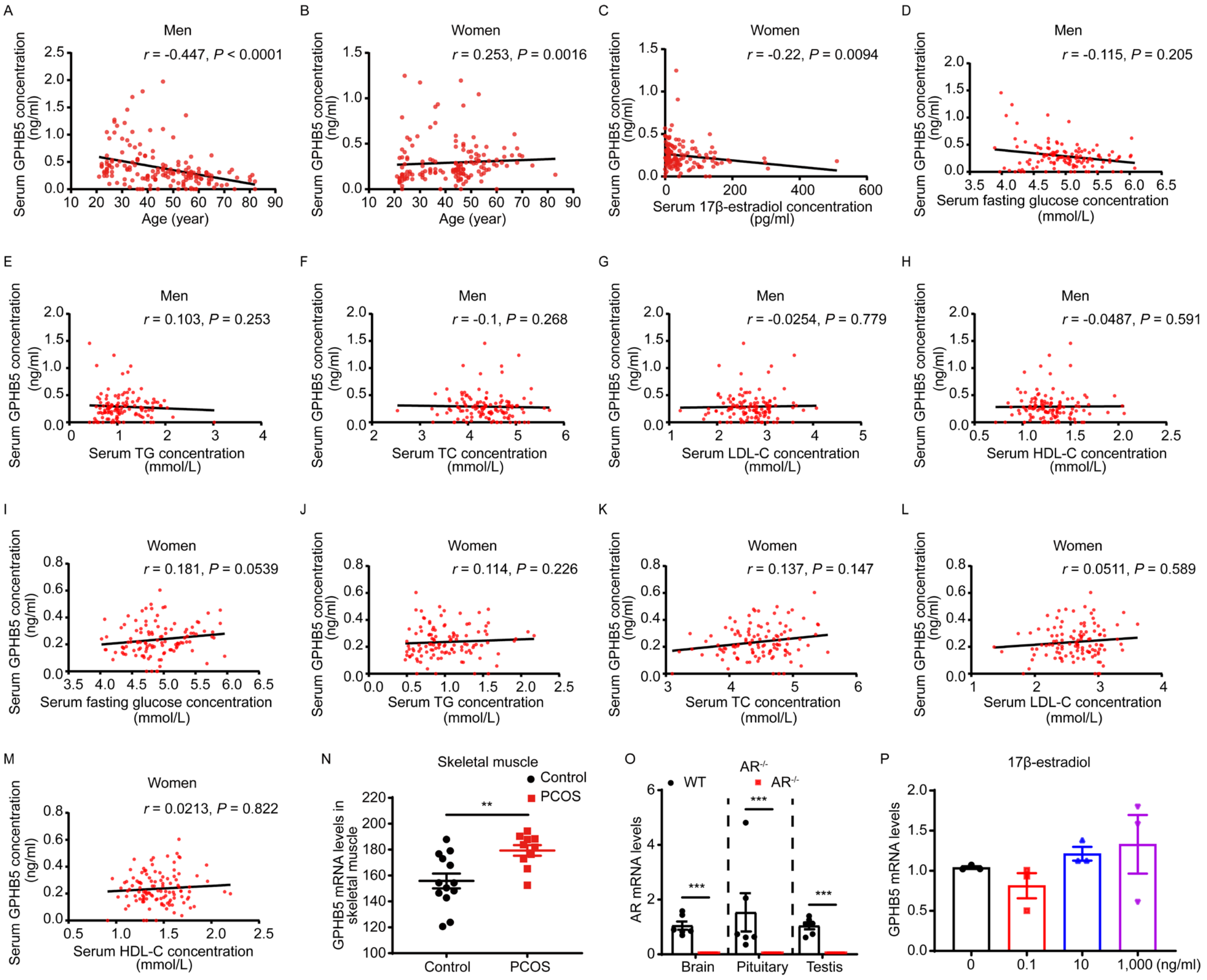

Supplemental Figure 1.

Assay of GPHB5 with age, 17β-estradiol, testosterone, fasting blood glucose, TG, TC, LDL-C and HDL-C. Correlation between age and GPHB5 levels in men (**A**) and women (**B**). (**C**) Correlation between 17β-estradiol and GPHB5 levels in women. Correlation between serum GPHB5 levels and fasting blood glucose (**D**), TG (**E**), TC (**F**), LDL-C (**G**), and HDL-C (**H**) levels in men. Correlation between serum GPHB5 levels and fasting blood glucose (**I**), TG (**J**), TC (**K**), LDL-C (**L**), and HDL-C (**M**) levels in women. (**N**) GPHB5 mRNA levels in skeletal muscle of control subjects and PCOS patients from GEO (accession numbers GSE8157). (**O**) AR mRNA levels in brain, pituitary and testis of mice (n = 6 for WT and n = 8 for $AR^{-/-}$ mice). (**P**) GPHB5 mRNA levels in C2C12 cells treated with 17β-estradiol for 24 h. All data represent means ± SEM; for correlation analyses, Spearman r and P values were reported (**A-M**); significant differences

were performed using unpaired two-tailed Student's t-test (**N**), Mann-Whitney U test (**O**) and one-way ANOVA with Dunnett's multiple comparisons test (**P**). $^{**}P < 0.01$, $^{***}P < 0.001$.

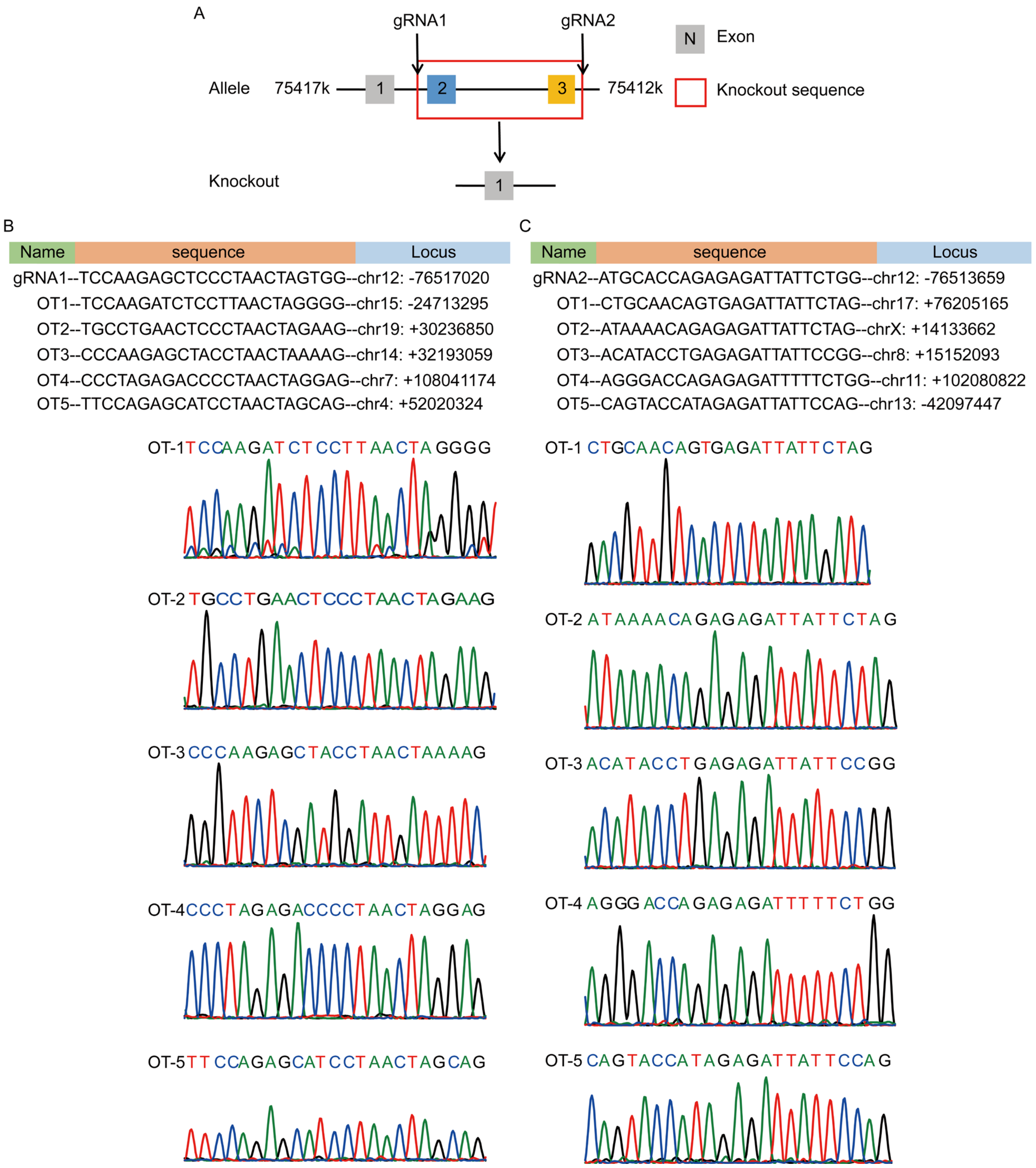

Supplemental Figure 2.

Analysis of the GPHB5 gene knockout. (**A**) Schematic diagram of GPHB5 gene knockout by CRISPR/Cas9. The possible off-target sites (OT) sequencing results in F2 generation mice (n = 13) for gRNA1 (**B**) and gRNA2 (**C**). There were no missed targets in any of the F2 generation mice, which indicates that their offspring will not miss them either.

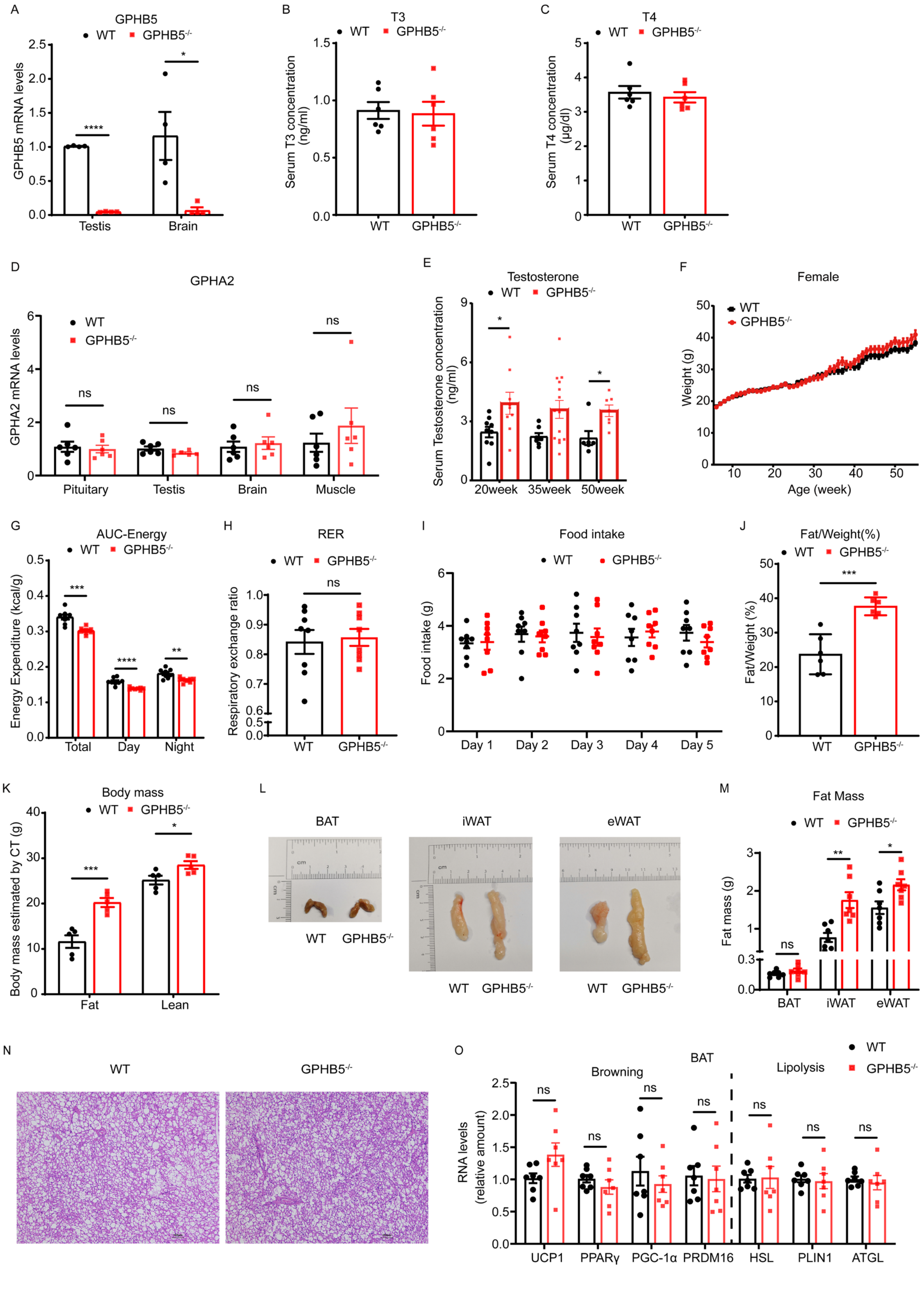
A
GPHB5
WT GPHB5-/-
GPHB5 mRNA levels
Testis
Brain
B
T3
Serum T3 concentration (ng/ml)
WT GPHB5-/-
C
T4
Serum T4 concentration (μg/dl)
WT GPHB5-/-
D
GPHA2
GPHA2 mRNA levels
Pituitary
Testis
Brain
Muscle
E
Testosterone
Serum Testosterone concentration (ng/ml)
20week 35week 50week
F
Female
Weight (g)
Age (week)
G
AUC-Energy
Energy Expenditure (kcal/g)
Total Day Night
H
RER
Respiratory exchange ratio
I
Food intake
Food intake (g)
Day 1 Day 2 Day 3 Day 4 Day 5
J
Fat/Weight(%)
Fat/Weight (%)
K
Body mass
Body mass estimated by CT (g)
Fat Lean
L
BAT
iWAT
eWAT
M
Fat Mass
Fat mass (g)
BAT iWAT eWAT
N
WT
GPHB5-/-
O
BAT
Browning
Lipolysis
RNA levels (relative amount)
UCP1 PPARγ PGC-1α PRDM16 HSL PLIN1 ATGL

Supplemental Figure 3.

Measurement of serum parameters and metabolism from GPHB5 knockout mice. (**A**) GPHB5 mRNA expression in mice testis and brain (*n* = 4 per group). Serum T3 (**B**) and T4 (**C**) levels of mice at 20 weeks of age (*n* = 6 per group). (**D**) GPHA2 mRNA expression in mice pituitary, testis, brain and muscle (*n* = 6 per group). (**E**) Testosterone levels in mice aged 20, 35 and 50 weeks (*n* = 6-9 for WT and *n* = 7-14 for $GPHB5^{-/-}$ mice). (**F**) Body weight curve of female mice (*n* = 16 for WT and *n* = 14 for $GPHB5^{-/-}$ mice). The weight of mice was tracked weekly from 6 to 55 weeks of age. Calculated AUC of energy expenditure (**G**) and average RER (**H**) over 24 h of mice (*n* = 8 per group). (**I**) Food intake of mice (*n* = 8 per group). (**J**) Fat/weight of mice (*n* = 6 per group) by Echo MRI. (**K**) Body fat mass and lean mass of mice (*n* = 5 per group) by micro-CT. (**L**) Representative photographs of BAT, iWAT and eWAT. (**M**) BAT, iWAT and eWAT mass of mice (*n* = 7 per group). (**N**) Representative images of H&E-stained BAT from mice after cold exposure (4°C, 24 h; *n* = 7 per group). Scale bars, 100 μm. (**O**) qPCR analysis of browning and lipolysis associated genes in BAT after cold exposure (4°C, 24 h; *n* = 7 per group). All data represent means ± SEM; significant differences were performed using unpaired two-tailed Student's t-test (**A**-**D**, **G**-**K**, **M**, **O**), Mann-Whitney U test (**E**) and two-way ANOVA with Sidak's multiple comparisons test (**F**). ns, not significant; $*P < 0.05$, $**P < 0.01$, $***P < 0.001$, $****P < 0.0001$.

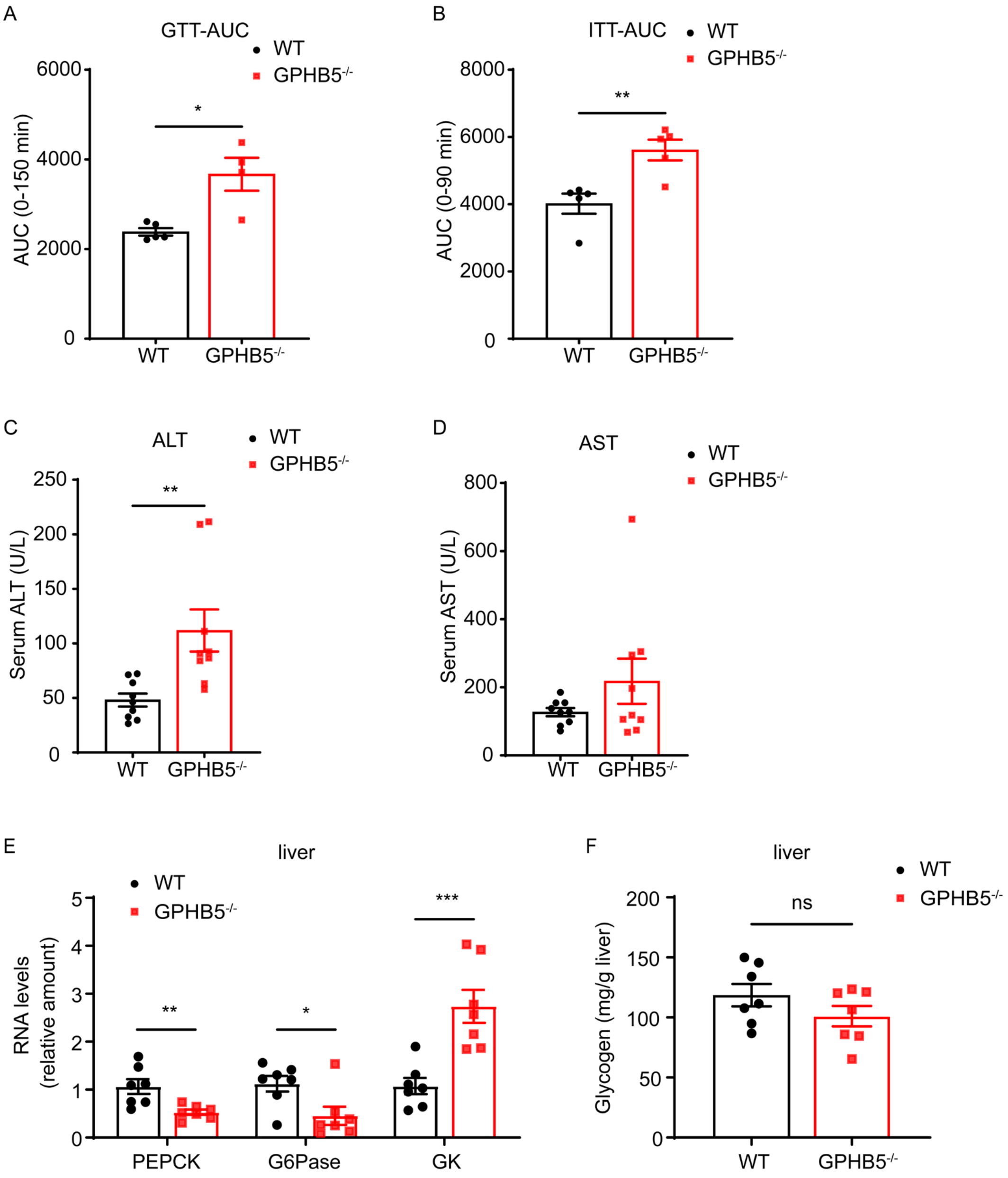

Supplemental Figure 4.

Lack of GPHB5 led to glucose intolerance, insulin resistance and impaired liver function in mice. Glucose tolerance test (**A**) and Insulin tolerance test (**B**) calculated AUC in mice (n = 4-5 per group). ALT (**C**) and AST (**D**) levels in serum of mice (n = 9 per group). (**E**) qPCR analysis of PEPCK, G6Pase and GCK mRNA expression in liver of mice (n = 7 per group). (**F**) Glycogen levels in liver of mice (n = 7 per group). All data represent means ± SEM; significant differences were performed using unpaired two-tailed Mann-Whitney U test (**A-D**) and Student's t-test (**E**, **F**). ns, not significant; *$P < 0.05$, **$P < 0.01$, ***$P < 0.001$.

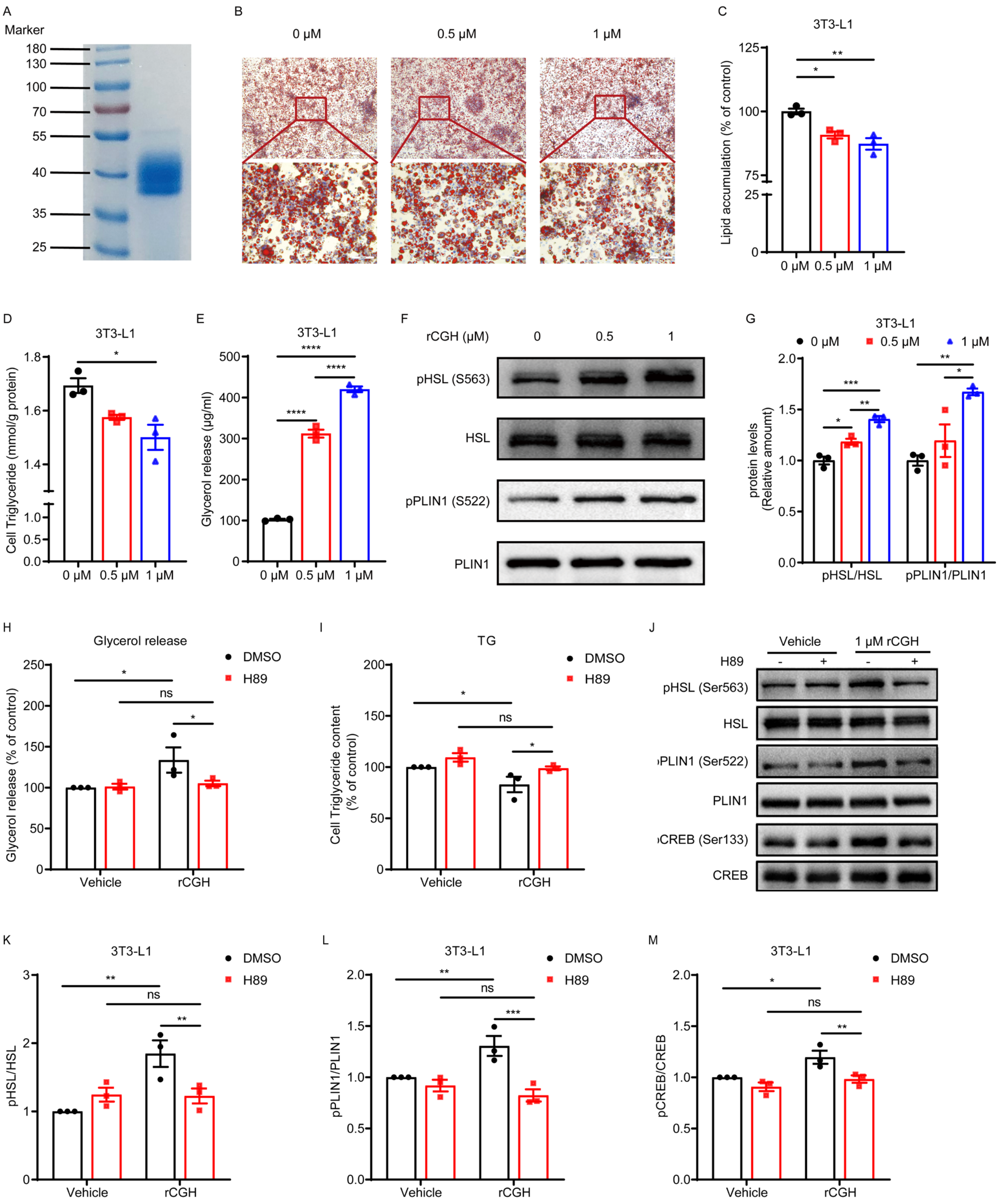
A
Marker
180
130
100
70
55
40
35
25
B
0 μM
0.5 μM
1 μM
C
3T3-L1
Lipid accumulation (% of control)
125
100
75
25
0
0 μM 0.5 μM 1 μM
D
3T3-L1
Cell Triglyceride (mmol/g protein)
1.8
1.6
1.4
1.0
0.5
0.0
0 μM 0.5 μM 1 μM
E
3T3-L1
Glycerol release (μg/ml)
500
400
300
200
100
0
0 μM 0.5 μM 1 μM
F
rCGH (μM)
0
0.5
1
pHSL (S563)
HSL
pPLIN1 (S522)
PLIN1
G
3T3-L1
0 μM
0.5 μM
1 μM
protein levels
(Relative amount)
2.0
1.5
1.0
0.5
0.0
pHSL/HSL
pPLIN1/PLIN1
H
Glycerol release
DMSO
H89
Glycerol release (% of control)
250
200
150
100
50
0
ns
Vehicle
rCGH
I
TG
DMSO
H89
Cell Triglyceride content
(% of control)
200
150
100
50
0
ns
Vehicle
rCGH
J
Vehicle
1 μM rCGH
H89
pHSL (Ser563)
HSL
PLIN1 (Ser522)
PLIN1
CREB (Ser133)
CREB
K
3T3-L1
DMSO
H89
pHSL/HSL
3
2
1
0
ns
Vehicle
rCGH
L
3T3-L1
DMSO
H89
pPLIN1/PLIN1
2.0
1.5
1.0
0.5
0.0
ns
Vehicle
rCGH
M
3T3-L1
DMSO
H89
pCREB/CREB
2.0
1.5
1.0
0.5
0.0
ns
Vehicle
rCGH

Supplemental Figure 5.

rCGH promoted lipolysis in differentiated 3T3-L1 adipocytes. (**A**) Coomassie Blue-stained analysis of rCGH. The lane 1 in gel was Protein Ladder, and the lane 2 was rCGH (40 kDa). Oil Red O staining images (**B**), lipid content (**C**), TG content (**D)** and glycerol release (**E**) of differentiated 3T3-L1 adipocytes incubated with 0 μM, 0.5 μM and 1 μM rCGH. Scale bars, 100 μm. Western blot analysis (**F**) and quantification (**G**) of pHSL, HSL, pPlin1 and Plin1 of differentiated 3T3-L1 adipocytes incubated with 0 μM, 0.5 μM or 1 μM rCGH. Glycerol release (**H**) and TG content (**I**) in differentiated 3T3-L1 adipocytes incubated with vehicle and 1 μM rCGH after pretreatment with DMSO control and the PKA inhibitor H89. Western blot analysis (**J**) and quantification of pHSL (**K**), pPlin1 (**L**) and pCREB (**M**). All data represent means ± SEM; significant differences were performed using one-way ANOVA with Tukey's multiple comparison test (**C**-**E**, **G**) and two-way ANOVA (**H**, **I**, **K-M**). ns, not significant; *$P < 0.05$, **$P < 0.01$, ***$P < 0.001$, ****$P < 0.0001$.

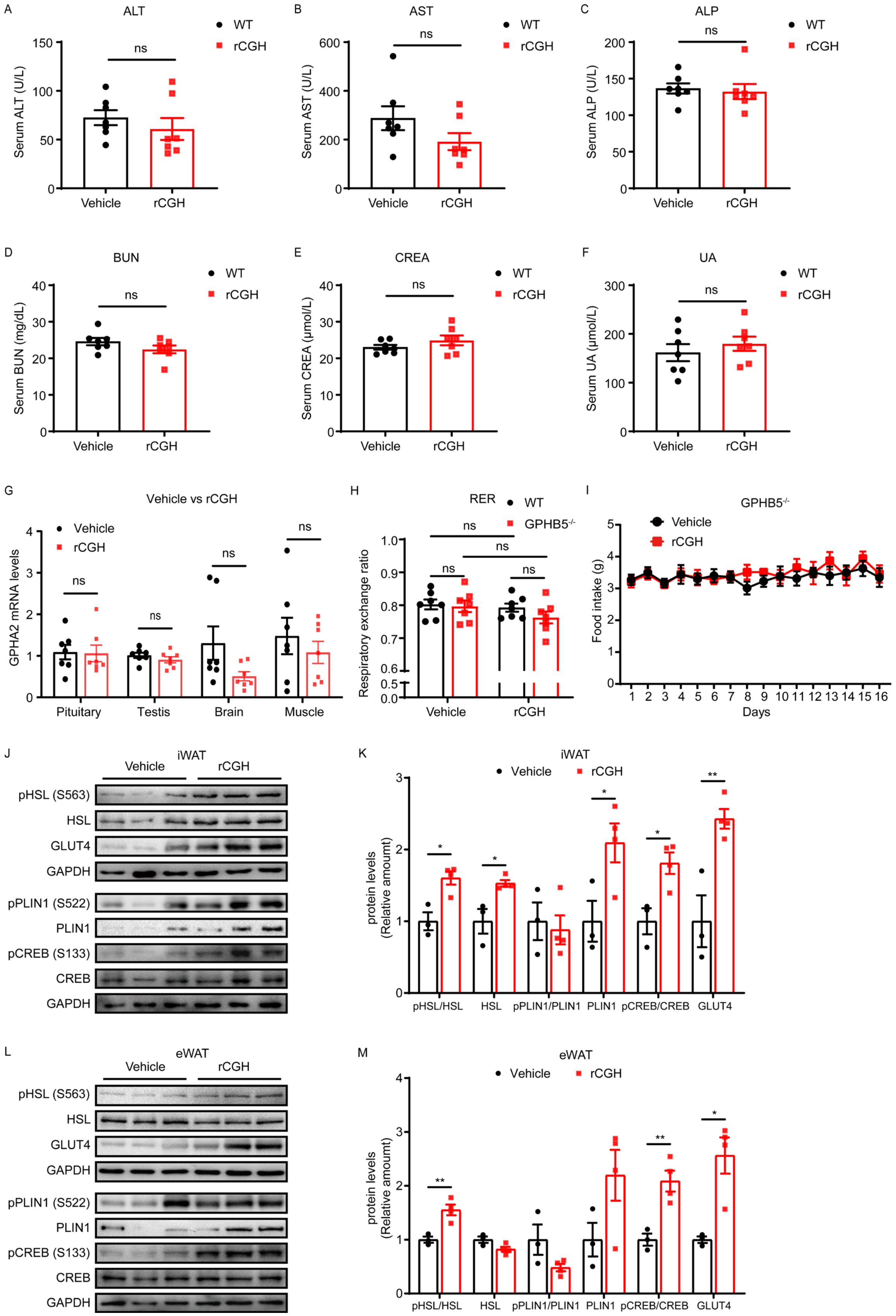
A
ALT
WT
rCGH
ns
Serum ALT (U/L)
Vehicle
rCGH
B
AST
Serum AST (U/L)
C
ALP
Serum ALP (U/L)
D
BUN
Serum BUN (mg/dL)
E
CREA
Serum CREA (μmol/L)
F
UA
Serum UA (μmol/L)
G
Vehicle vs rCGH
GPHA2 mRNA levels
Pituitary
Testis
Brain
Muscle
H
RER
GPHB5-/-
Respiratory exchange ratio
I
GPHB5-/-
Food intake (g)
Days
J
iWAT
pHSL (S563)
HSL
GLUT4
GAPDH
pPLIN1 (S522)
PLIN1
pCREB (S133)
CREB
K
protein levels
(Relative amount)
pHSL/HSL
pPLIN1/PLIN1
pCREB/CREB
L
eWAT
M

Supplemental Figure 6.

The effect of rCGH in GPHB5 deficiency mice. ALT (**A**), AST (**B**), ALP (**C**), BUN (**D**), CREA (**E**) and UA (**F**) levels in serum of WT mice treated with vehicle or 10 mg/kg rCGH (*n* = 7 per group). (**G**) GPHA2 mRNA expression in pituitary, testis, brain and muscle of WT mice treated with vehicle or 10 mg/kg rCGH (*n* = 7 per group). (**H**) Average RER over 24 h of WT and $GPHB5^{-/-}$ mice treated with vehicle or 10 mg/kg rCGH (*n* = 7 per group). (**I**) Food intake of $GPHB5^{-/-}$ mice treated with vehicle or 5 mg/kg rCGH (*n* = 7 per group). Western blot analysis of pHSL, HSL, pPlin1, Plin1, pCREB and Glut4 levels in iWAT (**J**, **K**) and eWAT (**L**, **M**) of $GPHB5^{-/-}$ mice treated with vehicle (*n* = 3) or 5 mg/kg rCGH (*n* = 4) for 16 days. All data represent means ± SEM; significant differences were performed using unpaired two-tailed Student's t-test (**A-G**, **K**, **M**) and two-way ANOVA with Sidak's multiple comparisons test (**H**, **I**). ns, not significant; $^{*}P < 0.05$, $^{**}P < 0.01$.

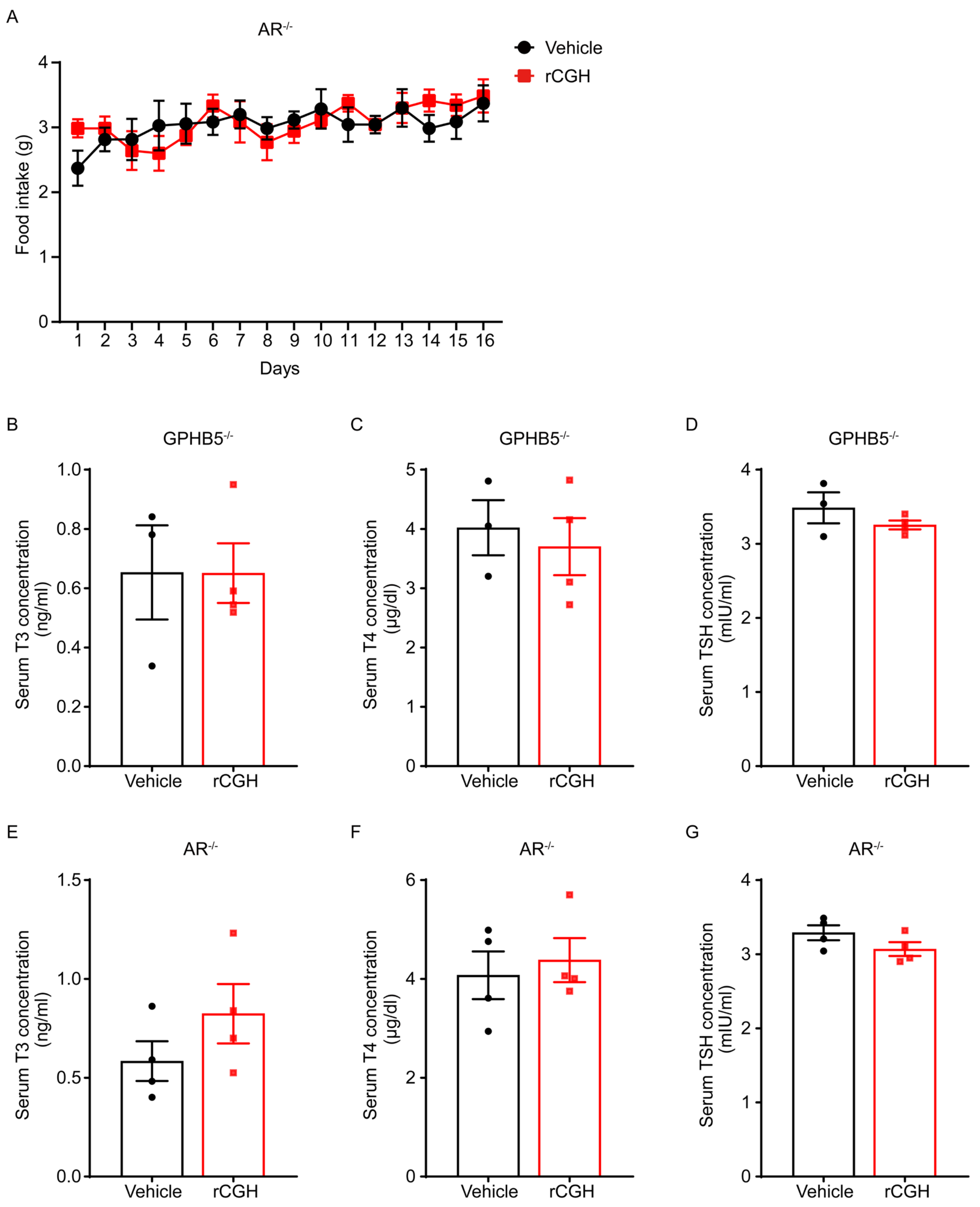

Supplemental Figure 7.

The changes of food intake in AR$^{-/-}$ mice and the thyroid-related hormones levels in GPHB5$^{-/-}$ or AR$^{-/-}$ mice following supplementation with rCGH. (**A**) Food intake of AR$^{-/-}$ mice treated with vehicle or 5 mg/kg rCGH (n = 7 per group). Serum T3 (**B**), T4 (**C**) and TSH (**D**) levels of

GPHB5$^{-/-}$ mice treated with vehicle (n = 3) or 5 mg/kg rCGH (n = 4) for 16 days. Serum T3 (**E**), T4 (**F**) and TSH (**G**) levels of AR$^{-/-}$ mice treated with vehicle or 5 mg/kg rCGH (n = 4 per group) for 16 days. All data represent means ± SEM; significant differences were performed using two-way ANOVA with Sidak's multiple comparisons test (**A**) and unpaired two-tailed Student's t-test (**B**-**G**).

Table S1.

Serum GPHB5 and testosterone levels in men and women at different ages.

Sex	Age (yr)	n (subjects)	GPHB5 (ng/ml)	Testosterone (ng/ml)
Men	21-30	34	0.434 (0.314-0.631)	6.90 (4.59-8.79)
	31-40	37	0.432 (0.196-0.710)	5.54 (4.07-7.67)
	41-50	34	0.308 (0.211-0.396)	4.07 (2.93-5.16)
	51-60	40	0.246 (0.108-0.320)	3.56 (2.78-6.13)
	61-70	16	0.189 (0.103-0.298)	3.90 (3.24-5.62)
	≥ 71	10	0.188 (0.062-0.274)	5.43 (3.91-8.93)
Women	21-30	39	0.164 (0.118-0.351)	0.280 (0.169-0.497)
	31-40	24	0.205 (0.180-0.314)	0.262 (0.178-0.513)
	41-50	51	0.213 (0.151-0.293)	0.250 (0.175-0.338)
	51-60	26	0.286 (0.216-0.370)	0.214 (0.150-0.336)
	≥ 61	12	0.360 (0.268-0.431)	0.380 (0.169-0.470)

Table S2.

The analyses of putative AR-binding sites of GPHB5 gene promoter.

Predicted sequence	Strand	Start	End	Score	Relative score
AAGGACATTCAGTGC	-	1261	1275	13.7223	0.926926
CAGCACTGAATGTCC	+	1259	1273	9.91637	0.875729
TGGAACATCGTGGTA	+	1445	1459	8.63162	0.858447

AAGAACAAAATATTT	-	1197	1211	7.4502	0.842554
GGGGACGGACTGCTC	-	732	746	6.53795	0.830283
AAGAACTTGGGGATT	+	538	552	5.89537	0.821639

Table S3.

Mouse PCR and qPCR primers.

Gene name	Forward primer (5'-3')	Reveser primer (5'-3')	Annealing Temperature	Product size
**mouse PCR primers**				
GPHB5-pair1	TAGCCACAACACACCCAGGACAC	CGGATGGGCATCCTTGTGAG	65 °C	WT: 3897 bp; MT: ~550 bp, delete~3340 bp
GPHB5-pair2	GTGACCCAAACCTCACACATCCACT	CGGATGGGCATCCTTGTGAG	65 °C	Heterozygote: 427 bp; Homozygote: no PCR bands
gRNA1-OT1	CAGCCCTCTTTATGACCATACAAC	GTTGACATCACTCTGCTGACTATCC	58 °C	619 bp
gRNA1-OT2	GTGATTCTGCCTGGTGTCCC	TCAAGATGGCGGCTAACCCT	58 °C	436 bp
gRNA1-OT3	GCTTTCTTCCTGATCTGTTCTGG	CCTATTCCCACCAGAACTCTACCTT	58 °C	630 bp

gRNA1-OT4	TACATAGCCTGGAATGCCTCG	CTTGTCTGTCACTGGCCGAAT	58 ℃	448 bp
gRNA1-OT5	AACATGAGTGGTATTCAGCAGAGG	AGTTAGCTTCATTTCTGGCACCT	58 ℃	655 bp
gRNA2-OT1	GTAACTGTCAGAAACTTGGCATATC	GCGGCATGTAGGAATTATAGC	58 ℃	552 bp
gRNA2-OT2	CAGAAGGTGTTGGACCCCCT	TCCAGTTCTTGCAGTACAGTTCAC	58 ℃	577 bp
gRNA2-OT3	TGTGATGCAGGTGTGAATTTGAG	GGCTCTTACAAATTACTGCTGGACT	58 ℃	624 bp
gRNA2-OT4	CCTGGTCTAACCTCGCACCT	GAGTGACTTCCGTCTCGCTGT	58 ℃	384 bp
gRNA2-OT5	GTTCTGCTCACACACTGGATGTCT	TCTCGTCAGCGACCTGACCTC	58 ℃	649 bp
mAR	AGGGACACTGAGAGACTCAAGAAG	CATAGTCAGGGTGTTCTCTCATGC	60 ℃	554 bp
mSRY	GACATCACTGGTGAGCATACACC	TCTCCTTCCTTACACACTACACATC	60 ℃	239bp
**mouse qPCR primers**				
β-actin	AAGTGTGACGTTGACATCCGTAAA	CAGCTCAGTAACAGTCCGCCTAGA	60 ℃	298 bp
GPB5	GTGGGAACCTGCACACTTT	GATGGTCTCACACTCAGTGG	60 ℃	311 bp
AR	GGACCATGTTTTACCCATCG	CCACAAGTGAGAGCTCCGTA	60 ℃	102 bp
UCP1	CTGCCAGGACAGTACCCAAG	TCAGCTGTTCAAAGCACACA	60 ℃	148 bp
PPARγ	GTACTGTCGGTTTCAGAAGTGCC	ATCTCCGCCAACAGCTTCTCCT	60 ℃	211 bp

PGC-1α	CCCTGCCATTGTTAAGACC	TGCTGCTGTTCCTGTTTTC	60 ℃	161 bp
PRDM16	CAGCACGGTGAAGCCATTC	GCGTGCATTCGCTTGTG	60 ℃	87 bp
HSL	AGGTGGGAATCTCTGCATCACTGT	TGTCCCTGAATAGGCACTGACACA	60 ℃	193 bp
PLIN1	GGCCTGGACGACAAAACC	CAGGATGGGCTCCATGAC	60 ℃	120 bp
ATGL	TGTGGCCTCATTCCTCCTAC	TCGTGGATGTTGGTGGAGCT	60 ℃	158 bp
GLUT1	TCAACGAGCATCTTCGAGAAGGCA	TCGTCCAGCTCGCTCTACAACAAA	60 ℃	120 bp
GLUT4	GGCTTTGTGGCCTTCTTTGAG	GACCCATAGCATCCGCAACAT	60 ℃	172 bp
Tbp	ACCCTTCACCAATGACTCCTATG	TGACTGCAGCAAATCGCTTGG	60 ℃	110 bp
FATP1	CGCTTTCTGCGTATCGTCTGCAAG	AAGATGCACGGGATCGTGTCT	60 ℃	90 bp
CD36	TCTTGGCTACAGCAAGGCCAGATA	AGCTATGCAGCATGGAACATGACG	60℃	173 bp
LPL	TGAGAAAGGGCTCTGCCTGA	GGGCATCTGAGAGCGAGTCTT	60 ℃	121 bp
DGAT1	CTCTGCCACAGCATTGAGAC	TGCTACGACGAGTTCTTGAG	60 ℃	221 bp
GPAT1	AGCAAGTCCTGCGCTATCAT	CTCGTGTGGGTGATTGTGAC	60 ℃	221 bp
GPAT3	CTTTGAAATCGGAGGAACCA	TTTGCAAACTGAACTGCGTC	60 ℃	198 bp

Table S4.

Change metabolites in iWAT.

Class	Metabolite	*P* value	Fold change	WT (nmol/g)	GPHB5$^{-/-}$ (nmol/g)
Fatty Acids	Myristoleic acid	0.0030	0.47	1.29	0.60
Fatty Acids	Myristic acid	0.0019	0.53	9.24	4.87
Fatty Acids	Pentadecanoic acid	0.028	0.52	3.18	1.66
Fatty Acids	alpha-Linolenic acid	0.020	0.54	37.03	20.12
Fatty Acids	gamma-Linolenic acid	0.025	0.57	3.42	1.95
Fatty Acids	10,13-Nonadecadienoic acid	0.042	0.59	1.41	0.84
Fatty Acids	10Z-Nonadecenoic acid	0.003	0.68	1.43	0.97
Amino Acids	Ornithine	0.0011	0.39	11.03	4.33
Amino Acids	beta-Alanine	0.028	0.41	4.02	1.65
Amino Acids	Serine	0.031	0.58	132.80	77.46
Amino Acids	Citrulline	0.038	0.38	6.67	2.51
Amino Acids	Proline	0.038	0.64	43.19	27.54
Amino Acids	Pyroglutamic acid	0.035	0.61	100.56	61.60
Amino Acids	4-Hydroxyproline	0.010	0.48	3.01	1.46
Carbohydrates	Glyceric acid	0.040	0.63	0.57	0.36
Carbohydrates	Glucose	0.0030	0.30	611.22	185.21
Carbohydrates	Fructose	0.00031	0.57	0.52	0.29
Carnitines	L-Carnitine	0.0053	0.61	33.22	20.22
Carnitines	Isovalerylcarnitine	0.017	0.37	0.11	0.039
Carnitines	Methylmalonylcarnitine	0.028	0.71	0.24	0.17
Phenylpropanoic Acids	2-Phenylpropionate	0.0047	0.30	0.052	0.016

Phenylpropanoic Acids	Hydrocinnamic acid	0.0019	0.11	0.088	0.0093
Bile Acids	CDCA (Chenodeoxycholic acid)	0.0083	1.76	0.11	0.20
Peptides	Carnosine	0.011	0.03	1.60	0.050
