# Supplementary material for: Reduced glycoprotein hormone β5 links male aging and testosterone decline to increased adiposity
Source: J Clin Invest. 2026 Feb 3;136(6):e192355. doi: 10.1172/JCI192355 (PMC12987616; doi:10.1172/JCI192355)

Full unedited gel for Figure 4C

UCP1

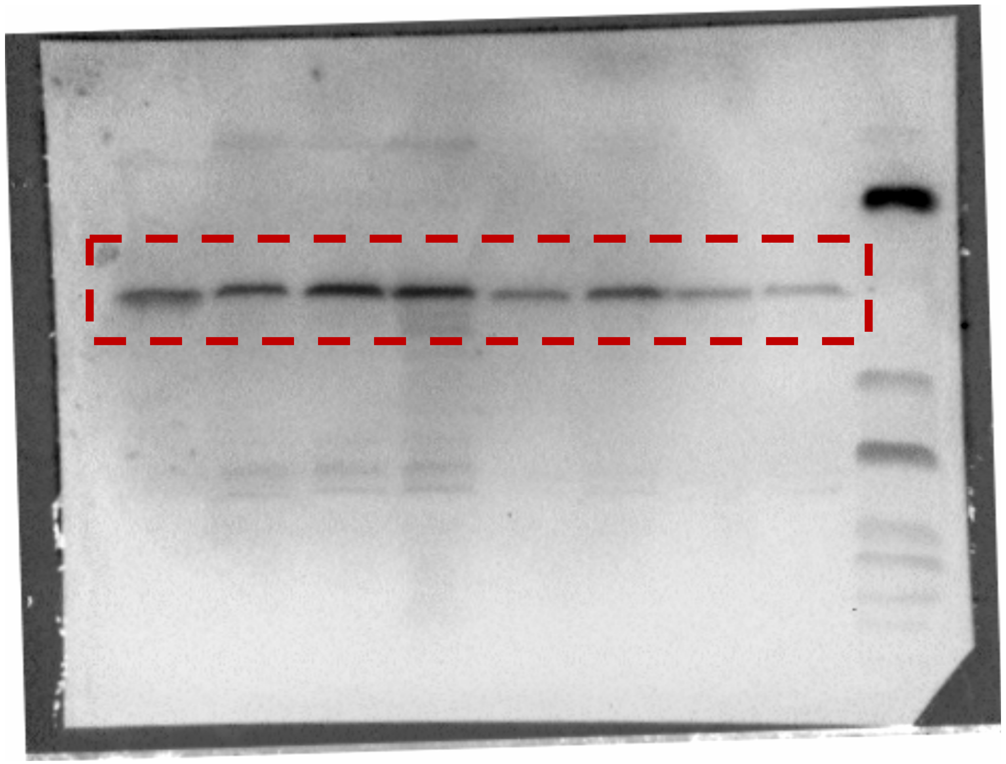

β-actin

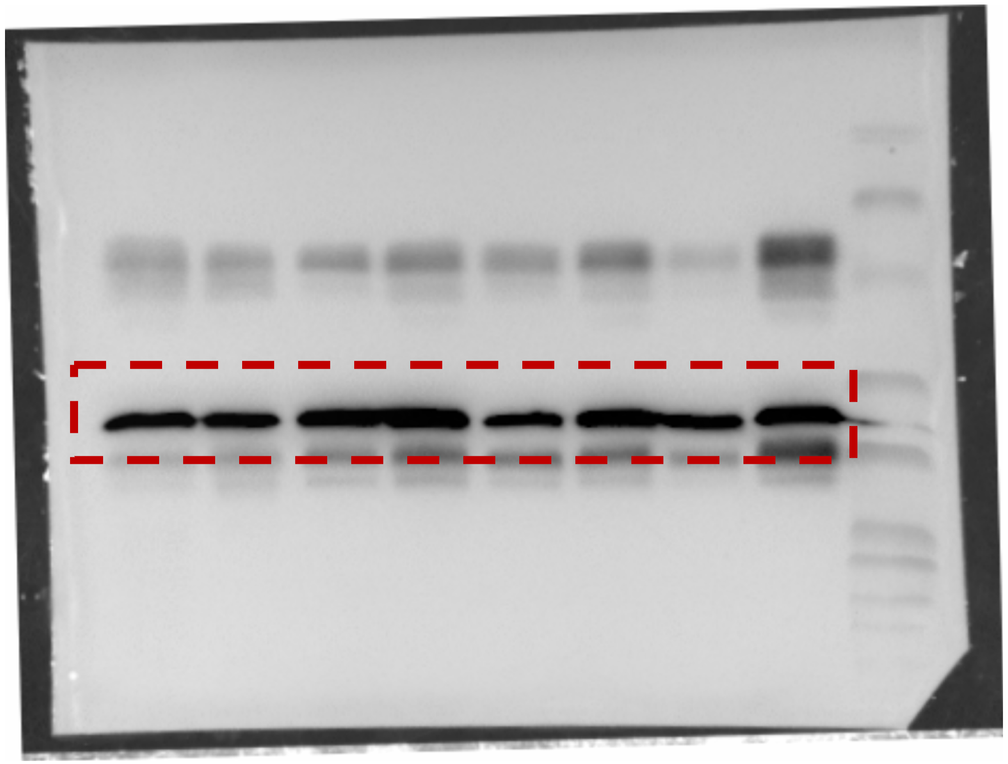

Full unedited gel for Supplemental Figure 5F

pHSL

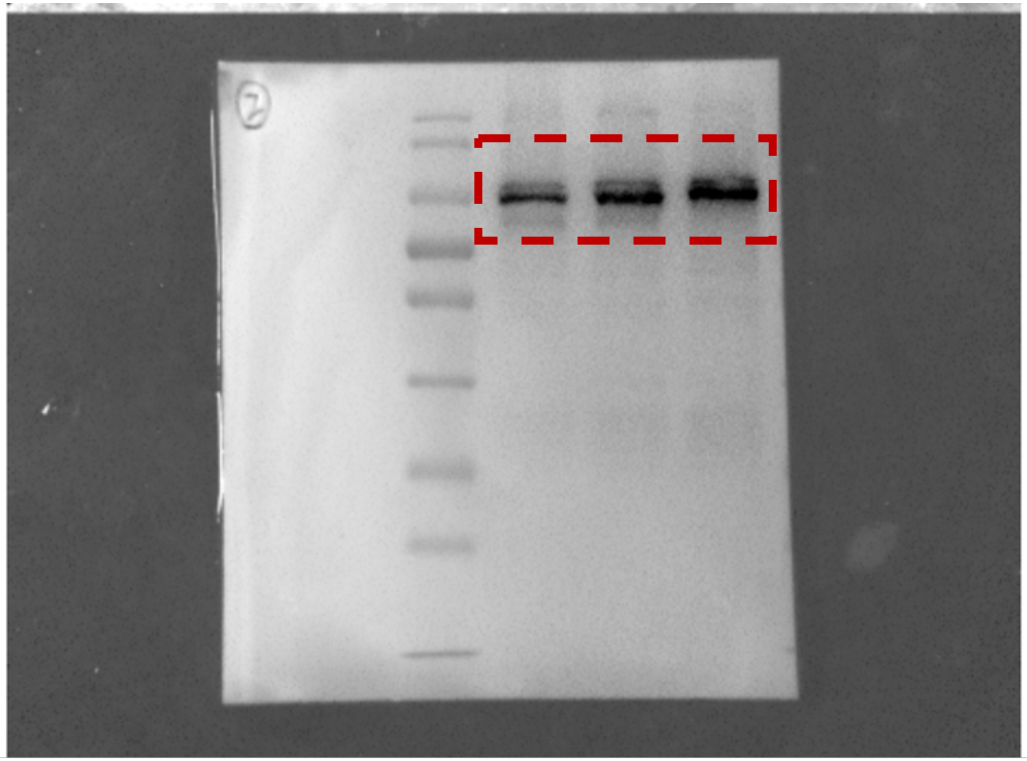

HSL

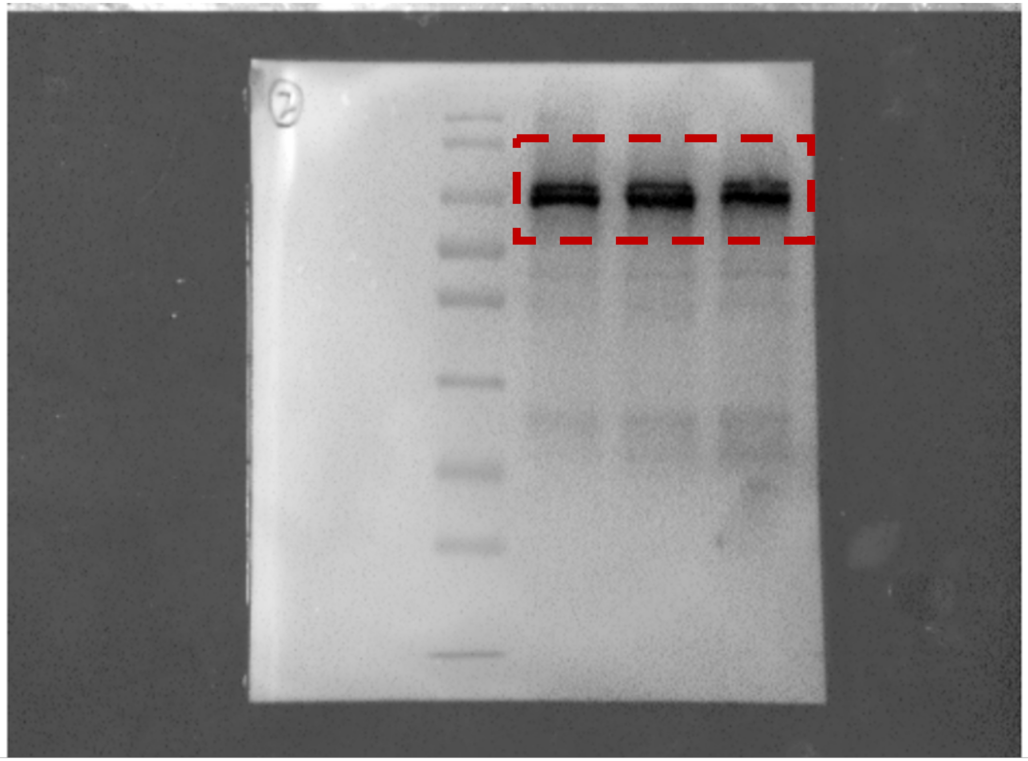

Full unedited gel for Supplemental Figure 5F

pPLIN1

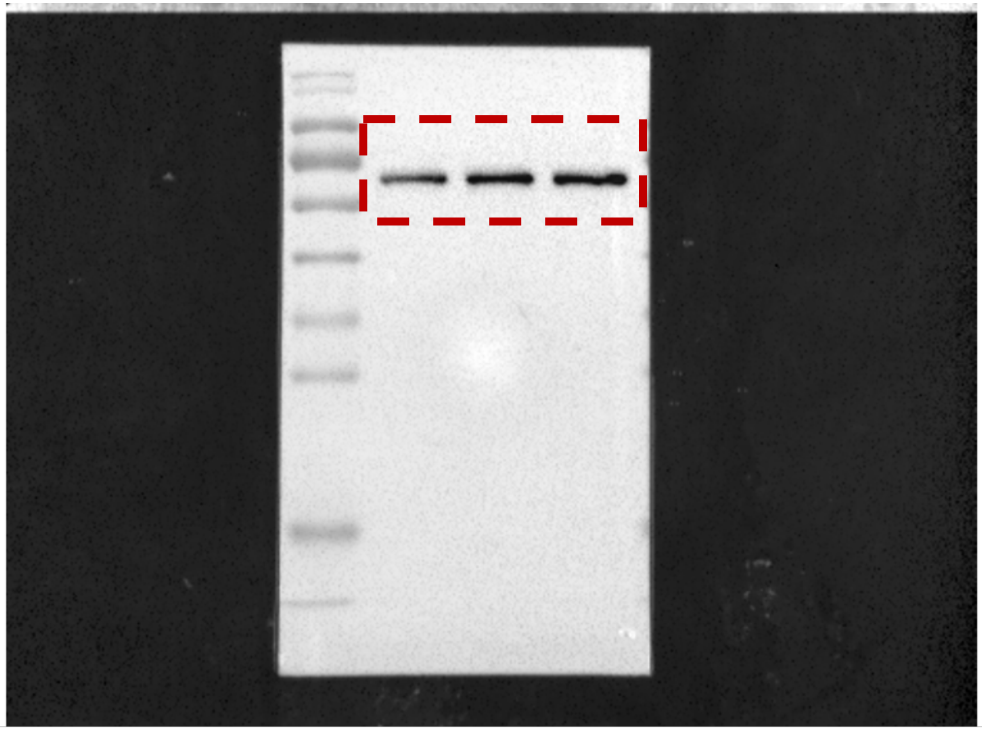

PLIN1

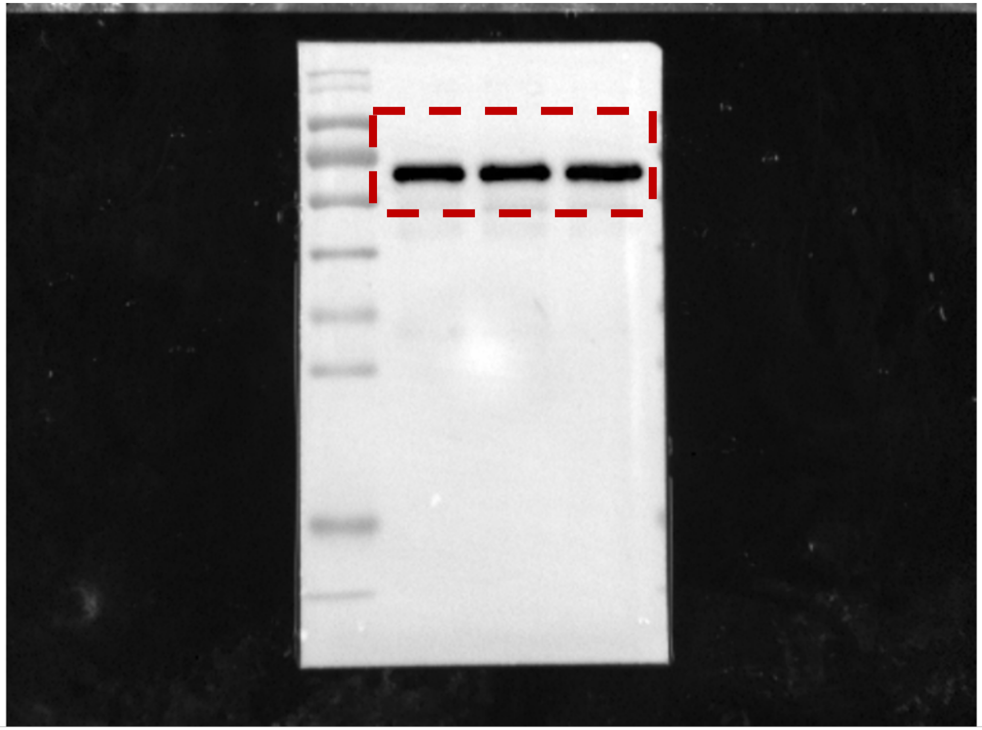

Full unedited gel for Supplemental Figure 5J

pHSL

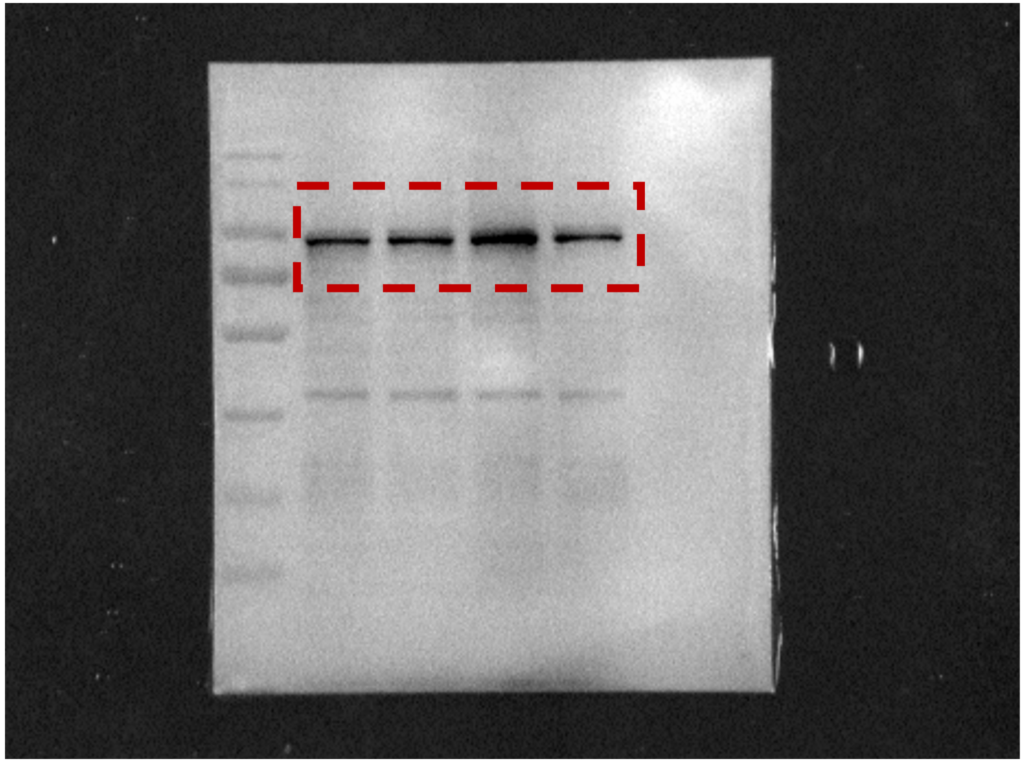

HSL

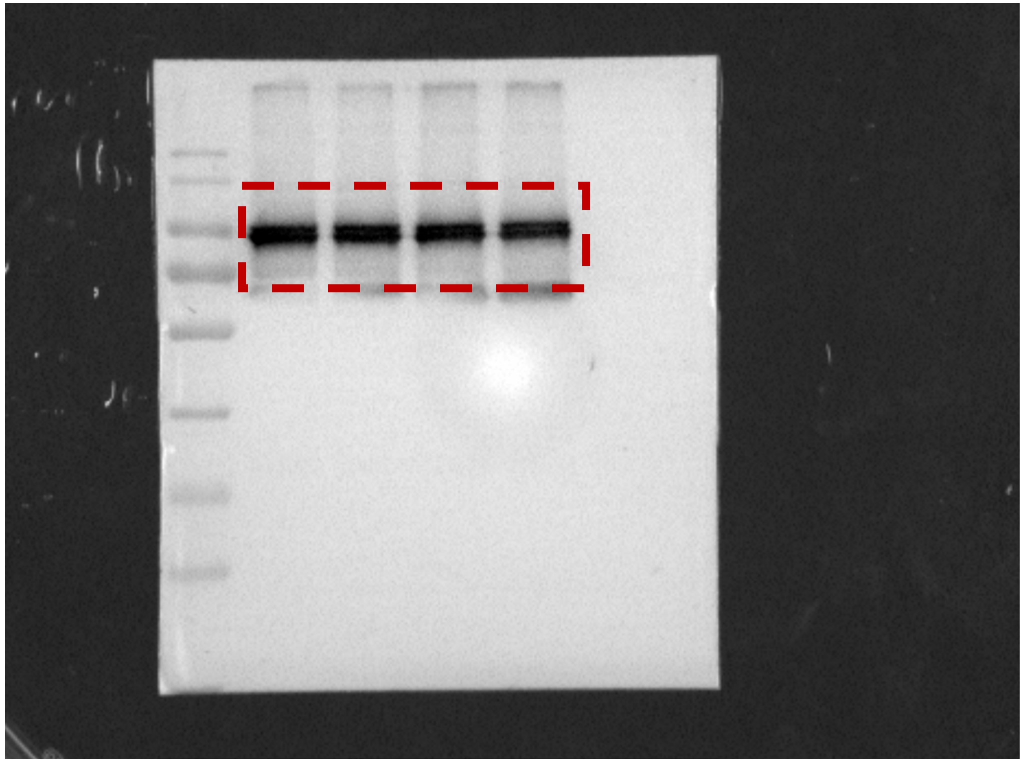

Full unedited gel for Supplemental Figure 5J

pPLIN1

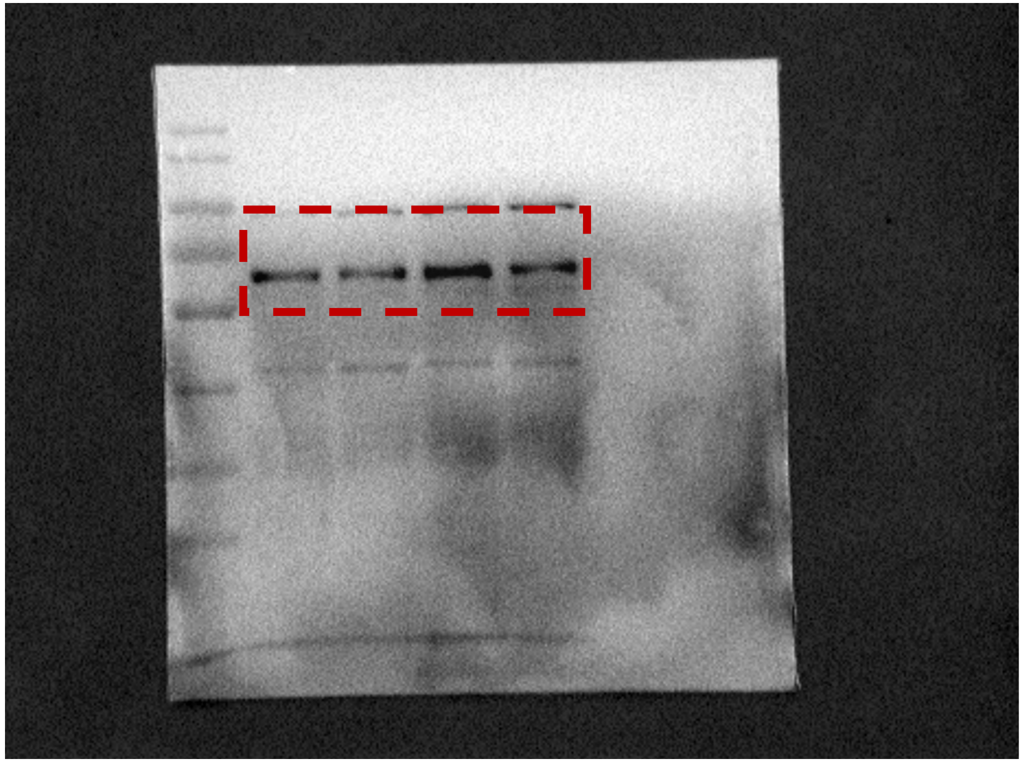

PLIN1

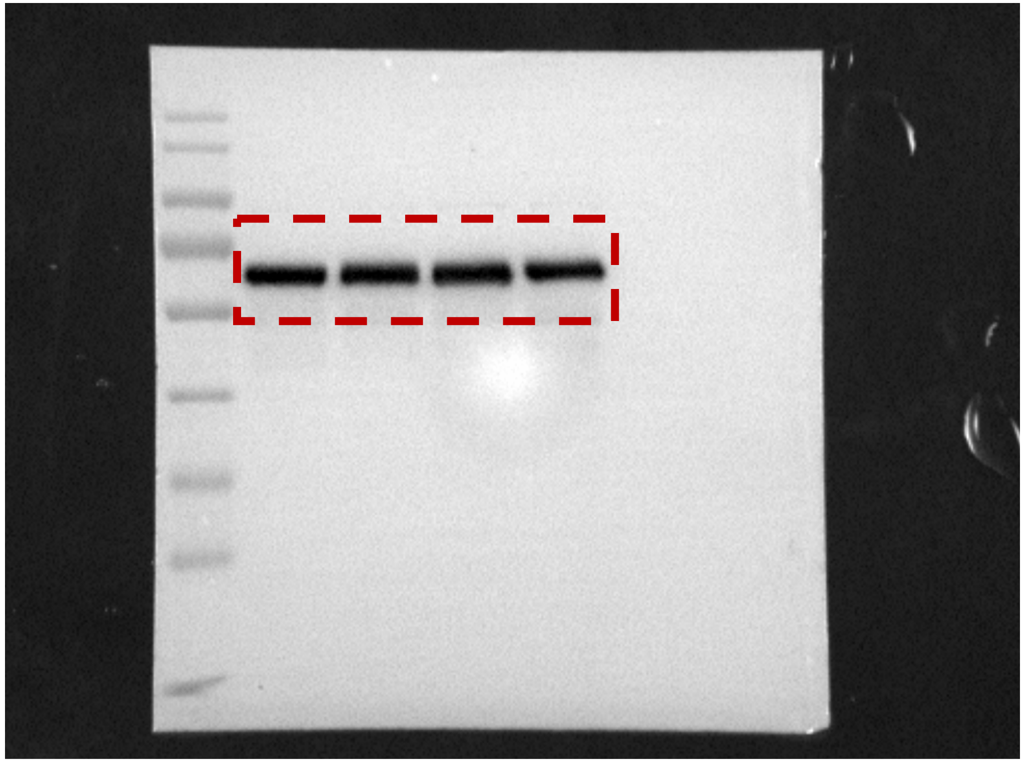

Full unedited gel for Supplemental Figure 5J

pCREB

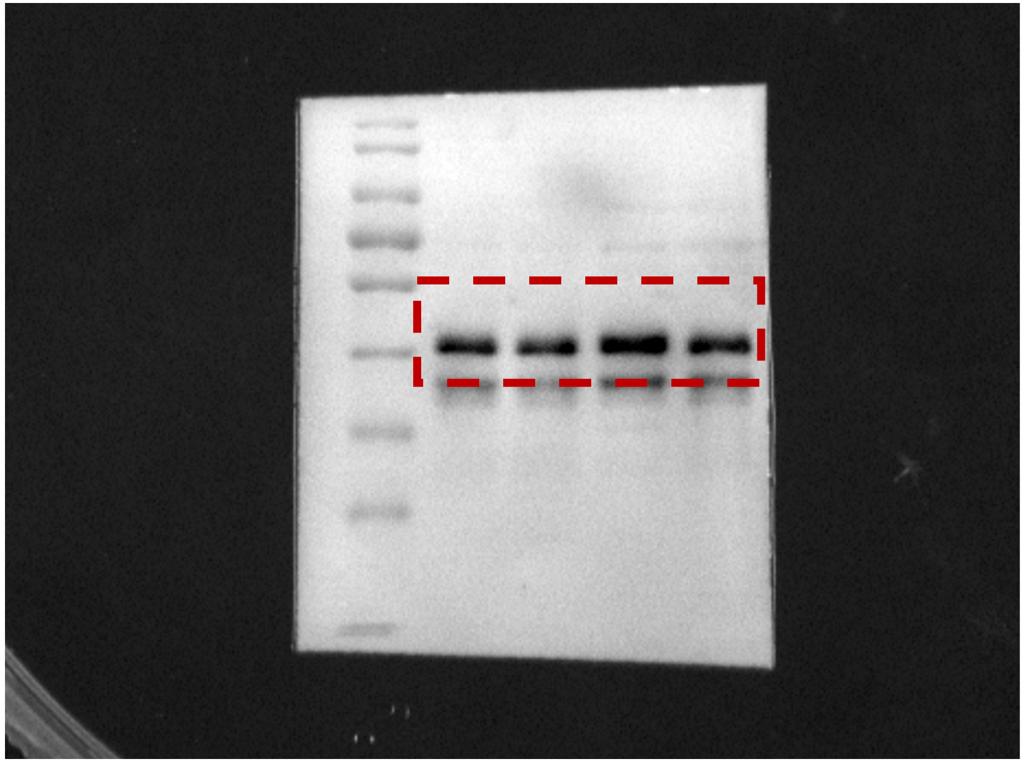

CREB

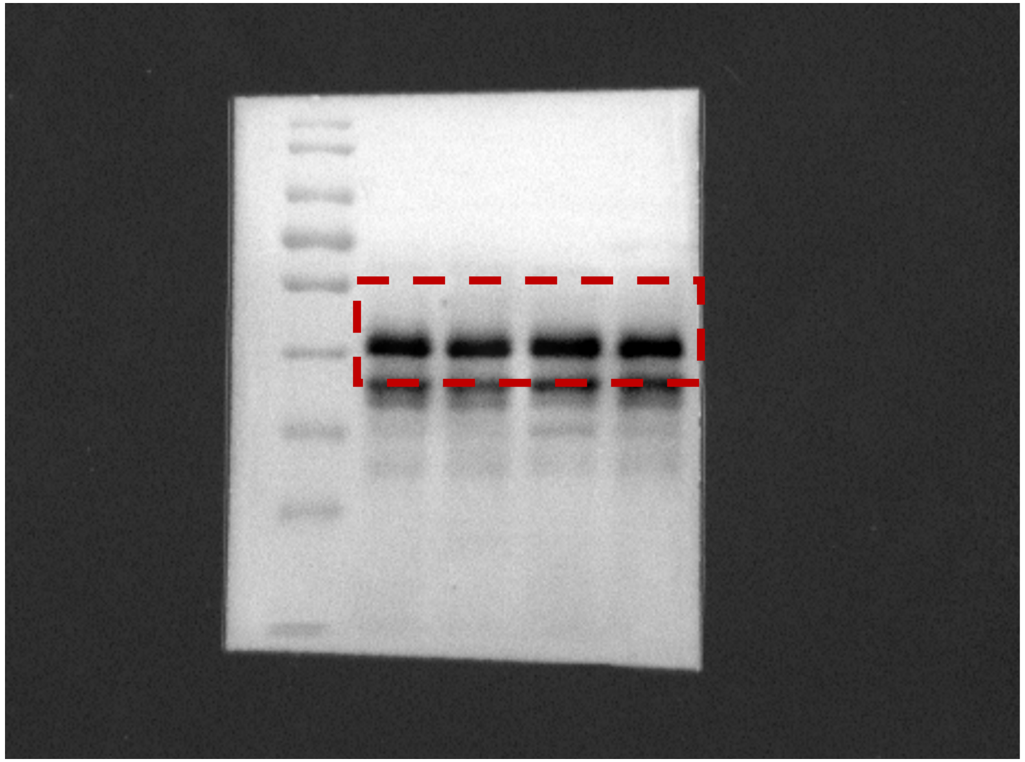

Full unedited gel for Supplemental Figure 6J

pHSL

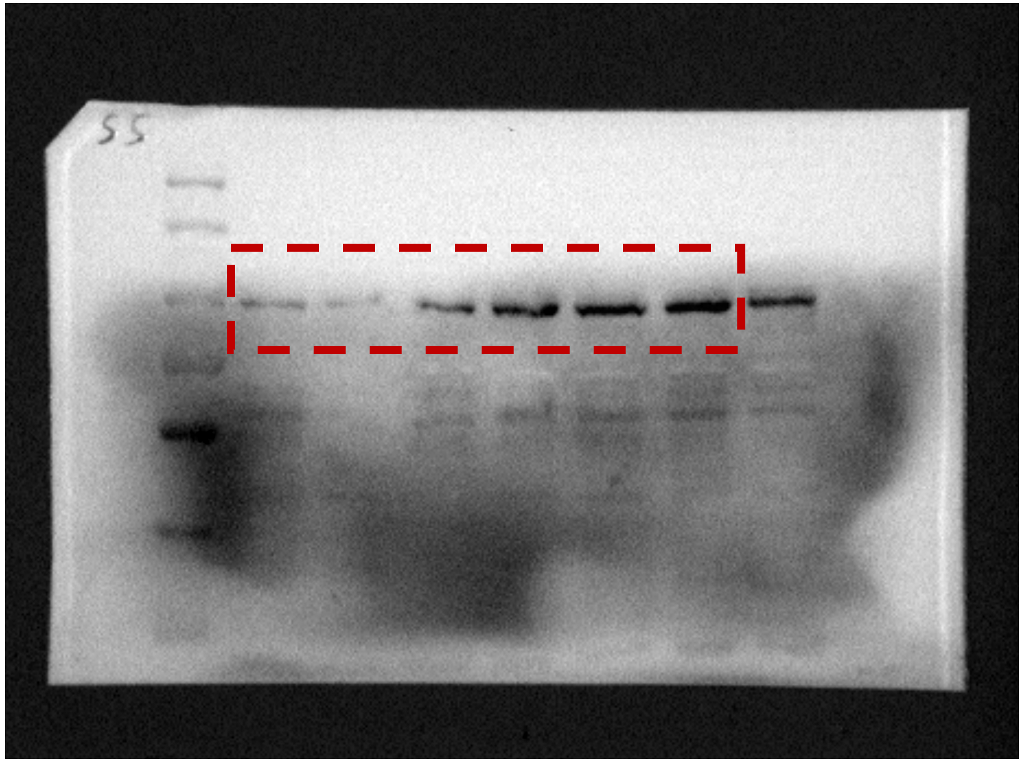

HSL

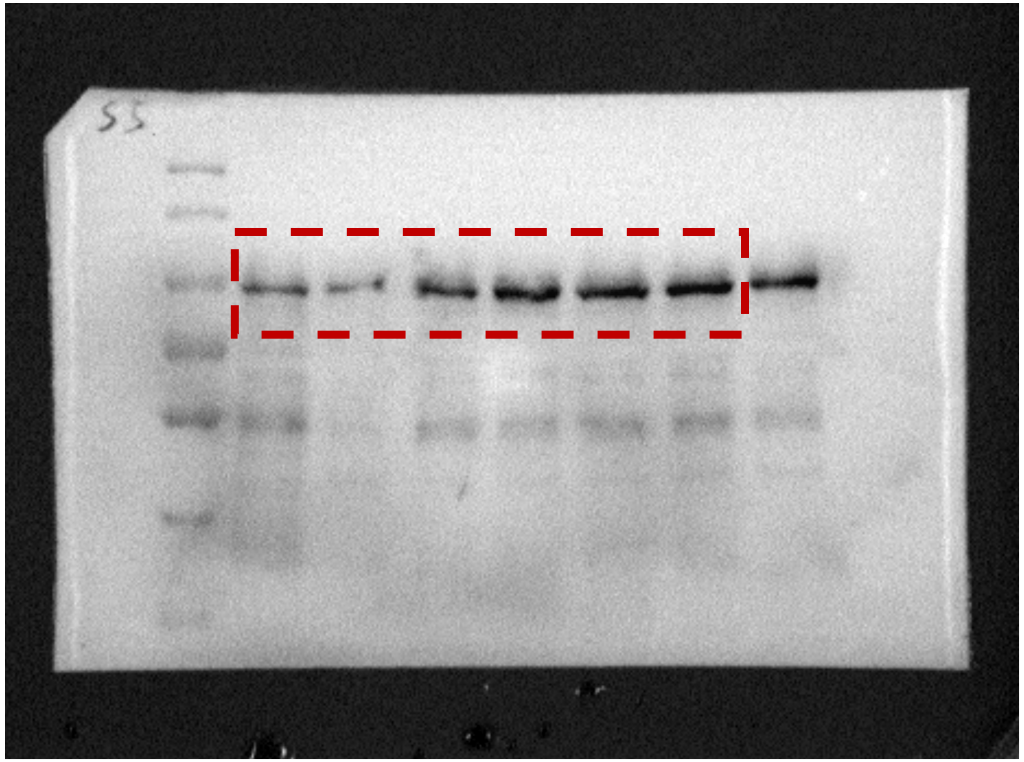

Full unedited gel for Supplemental Figure 6J

GLUT4

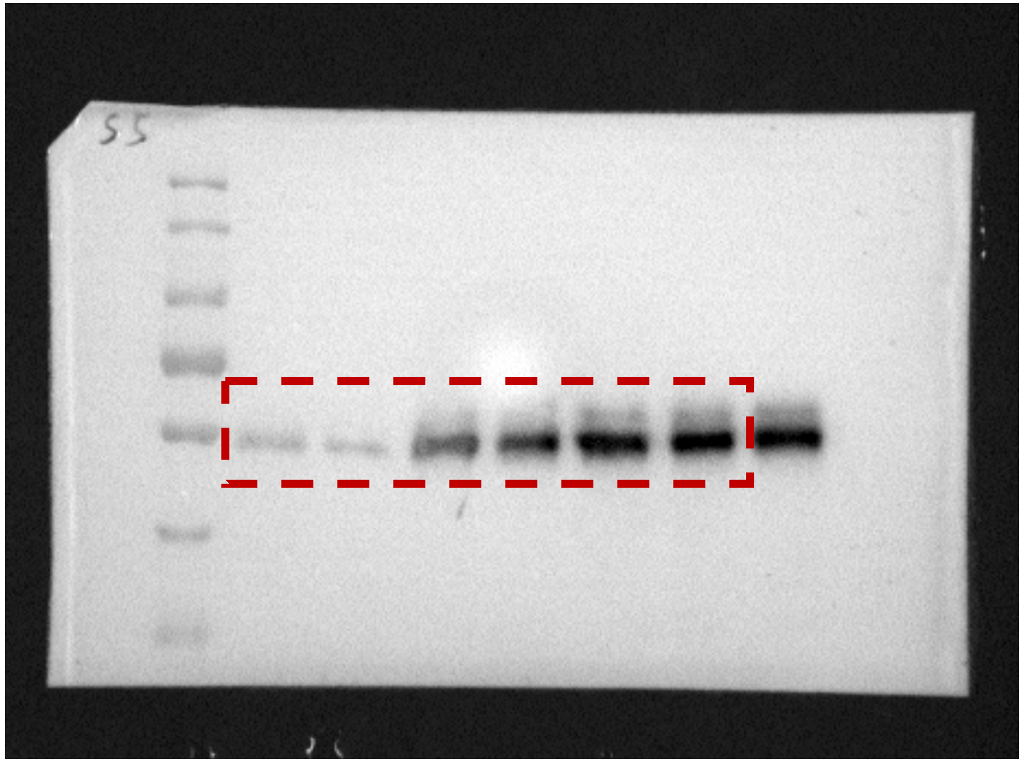

GAPDH

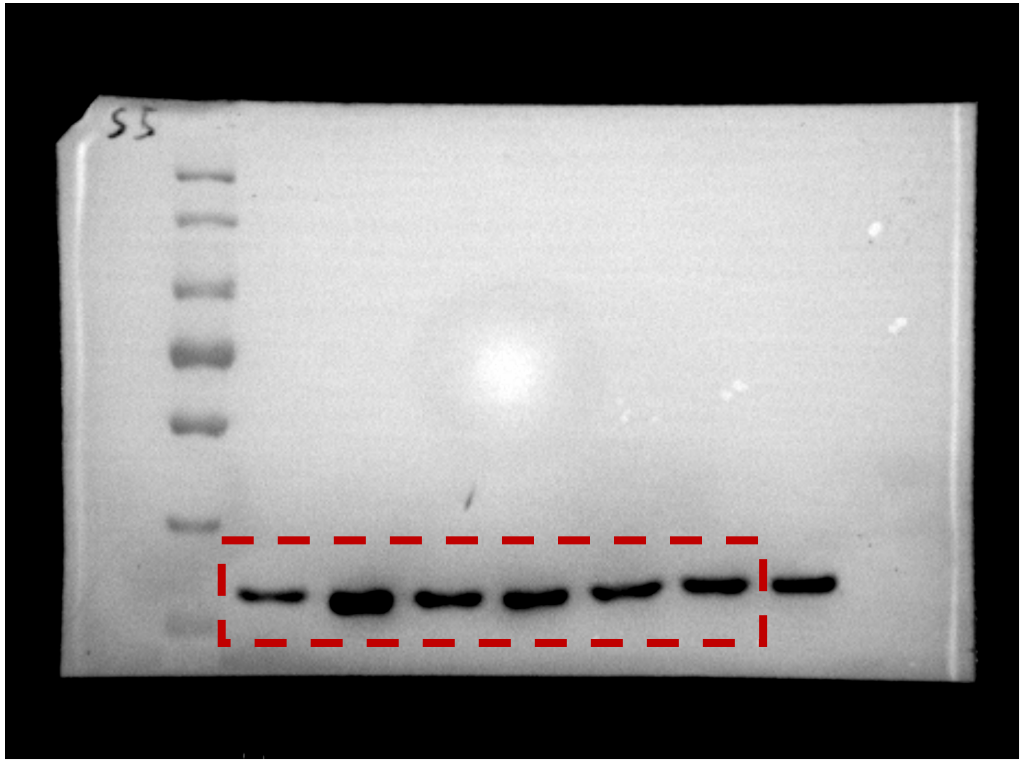

Full unedited gel for Supplemental Figure 6J

pPLIN1

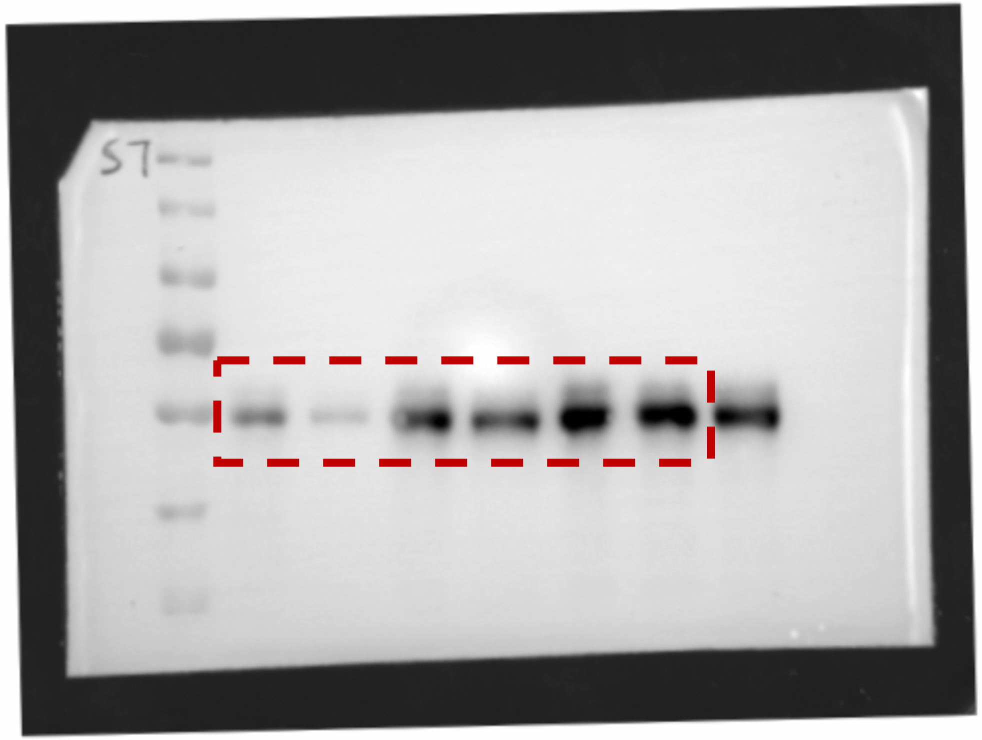

PLIN1

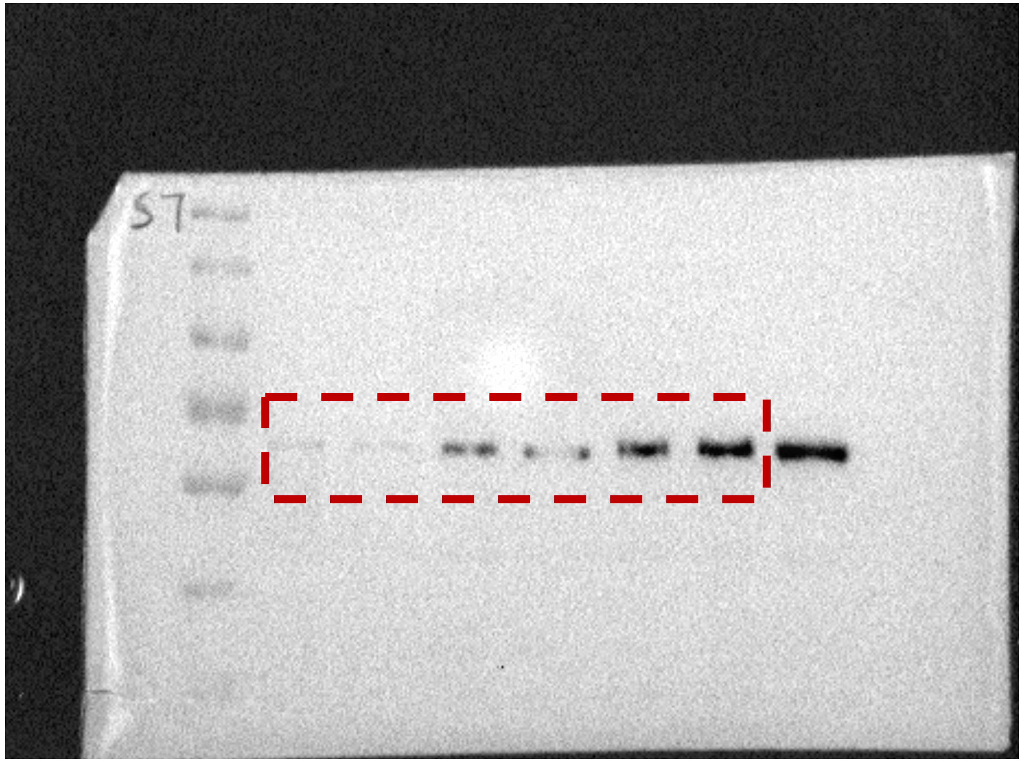

Full unedited gel for Supplemental Figure 6J

pCREB

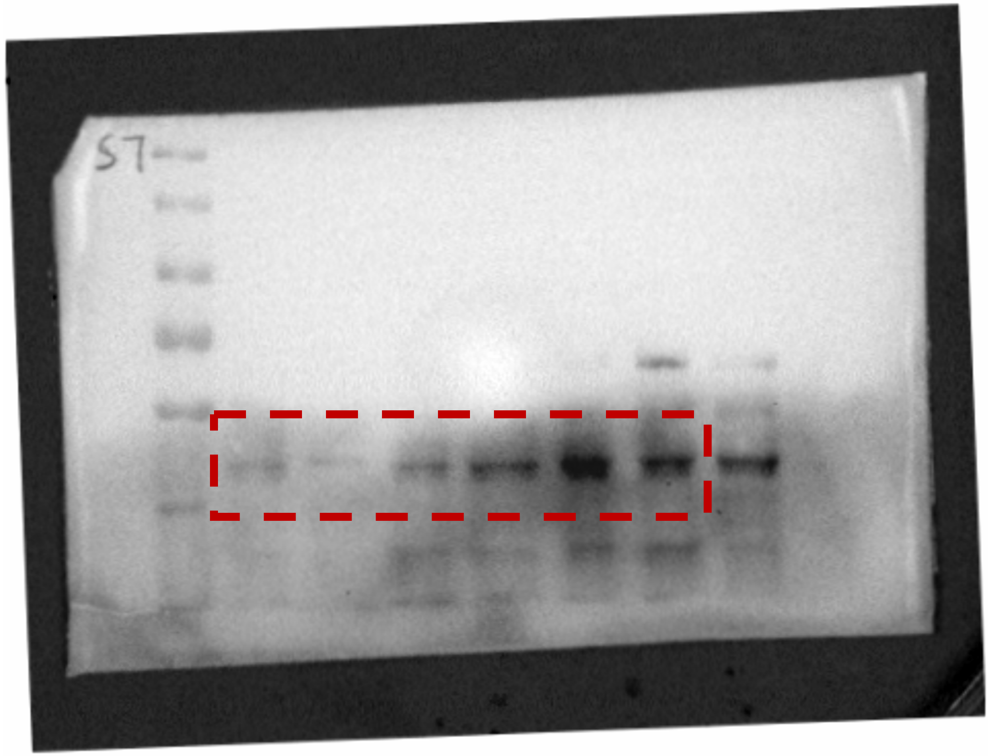

CREB

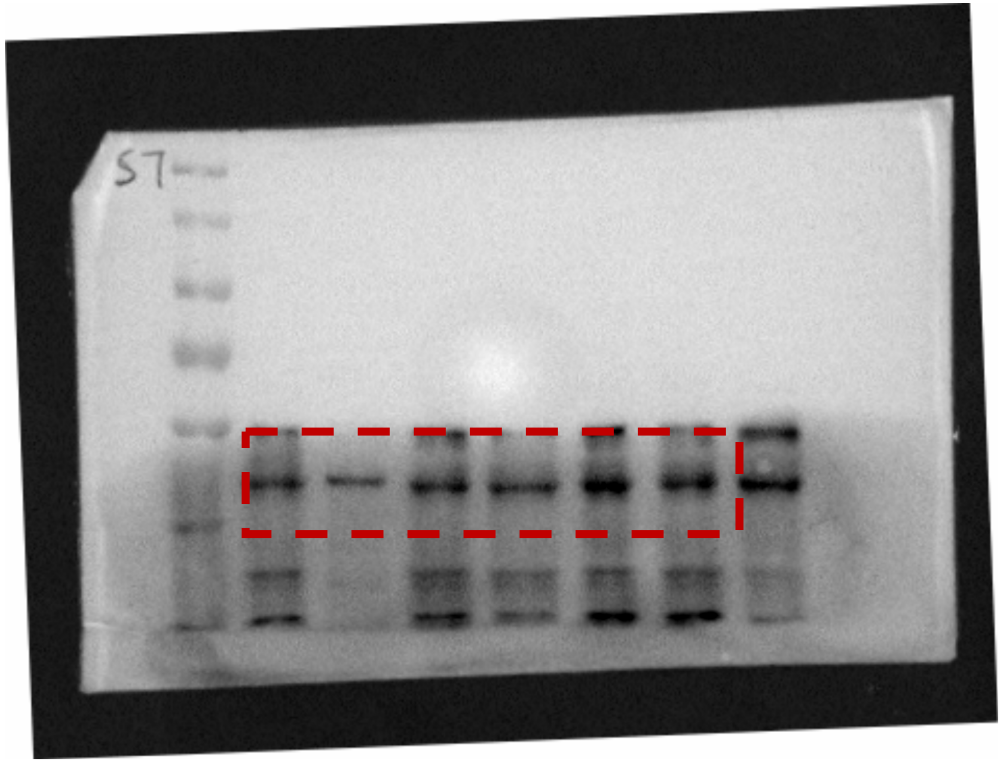

Full unedited gel for Supplemental Figure 6J

GAPDH

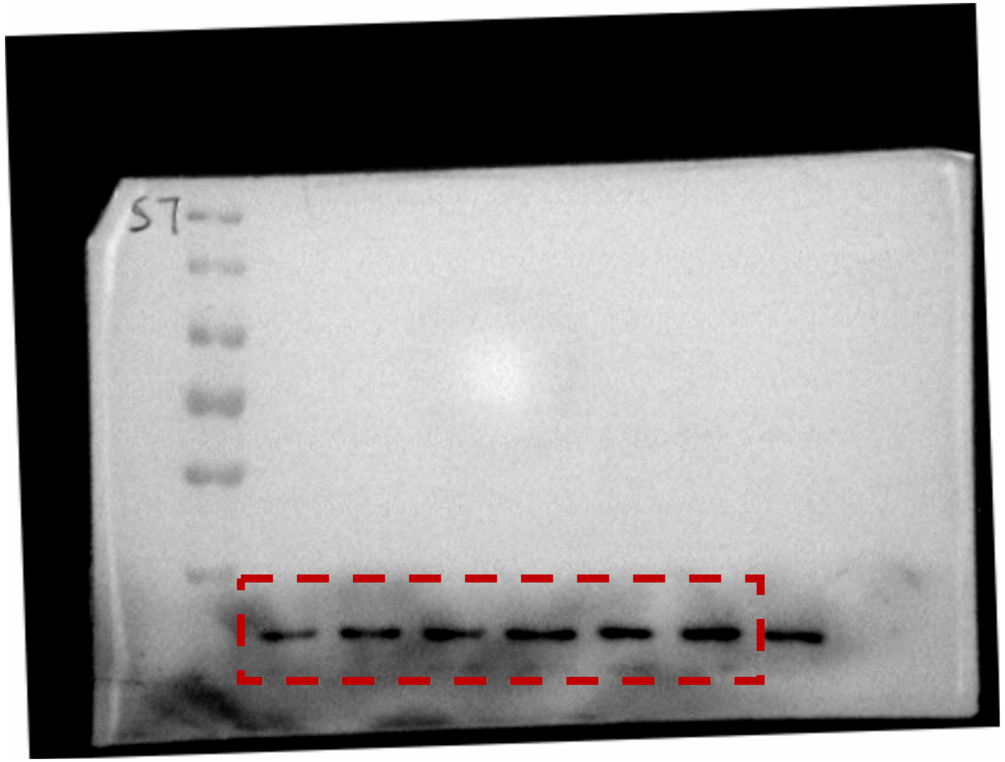

Full unedited gel for Supplemental Figure 6L

pHSL

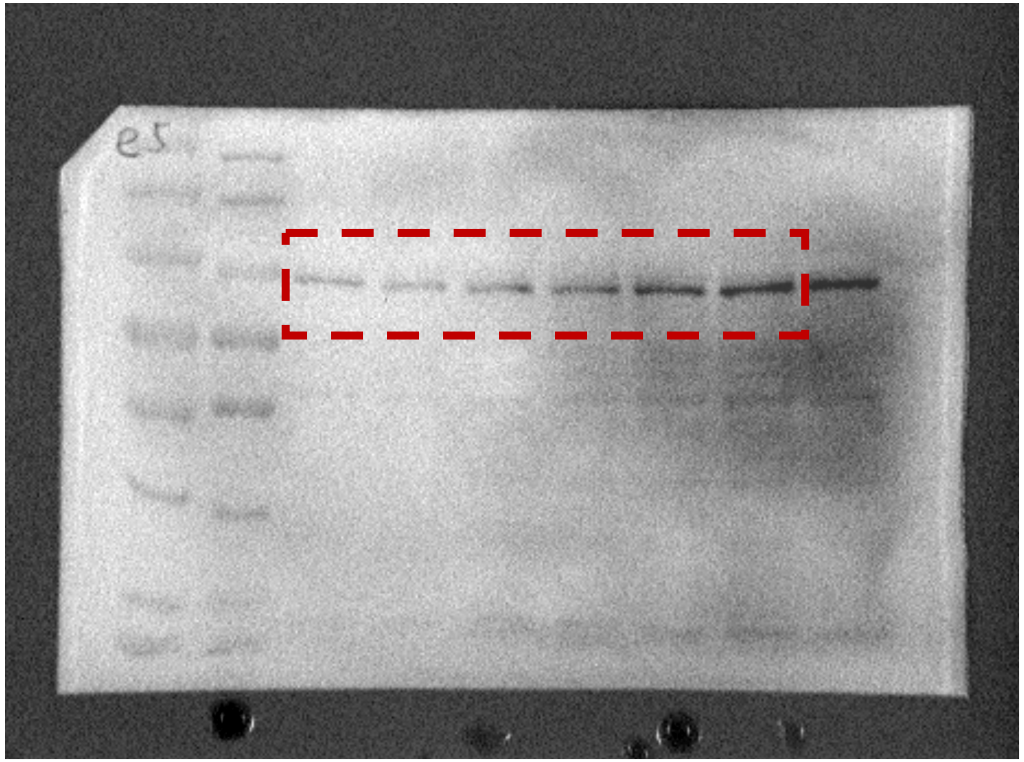

HSL

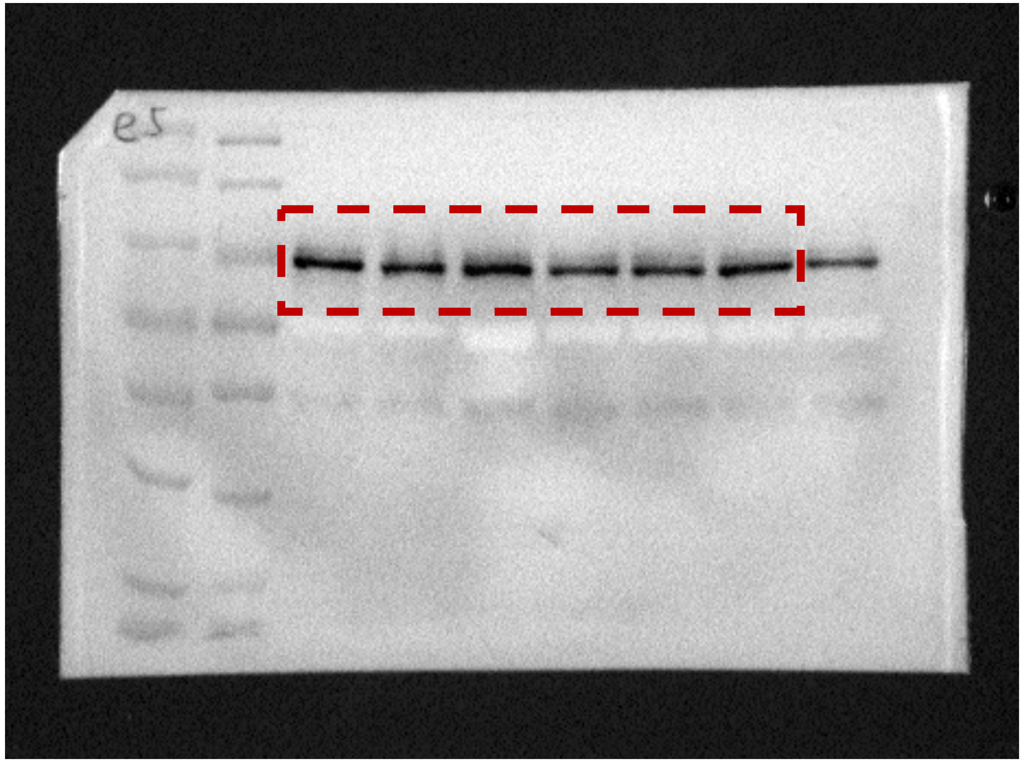

Full unedited gel for Supplemental Figure 6L

GLUT4

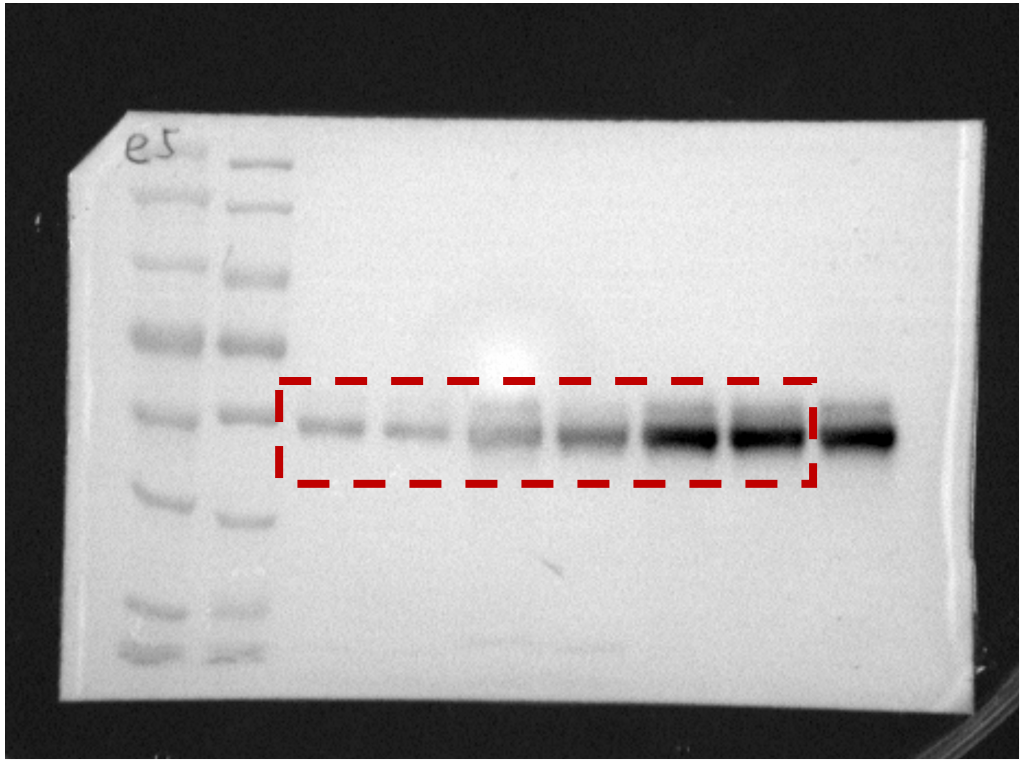

GAPDH

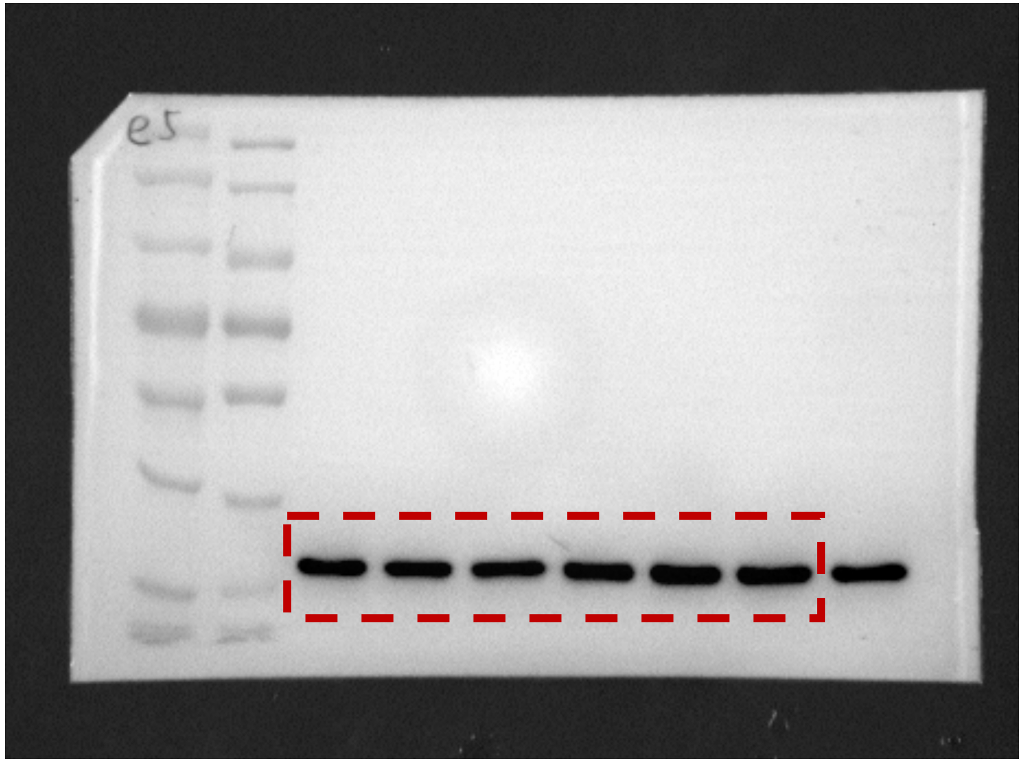

Full unedited gel for Supplemental Figure 6L

pPLIN1

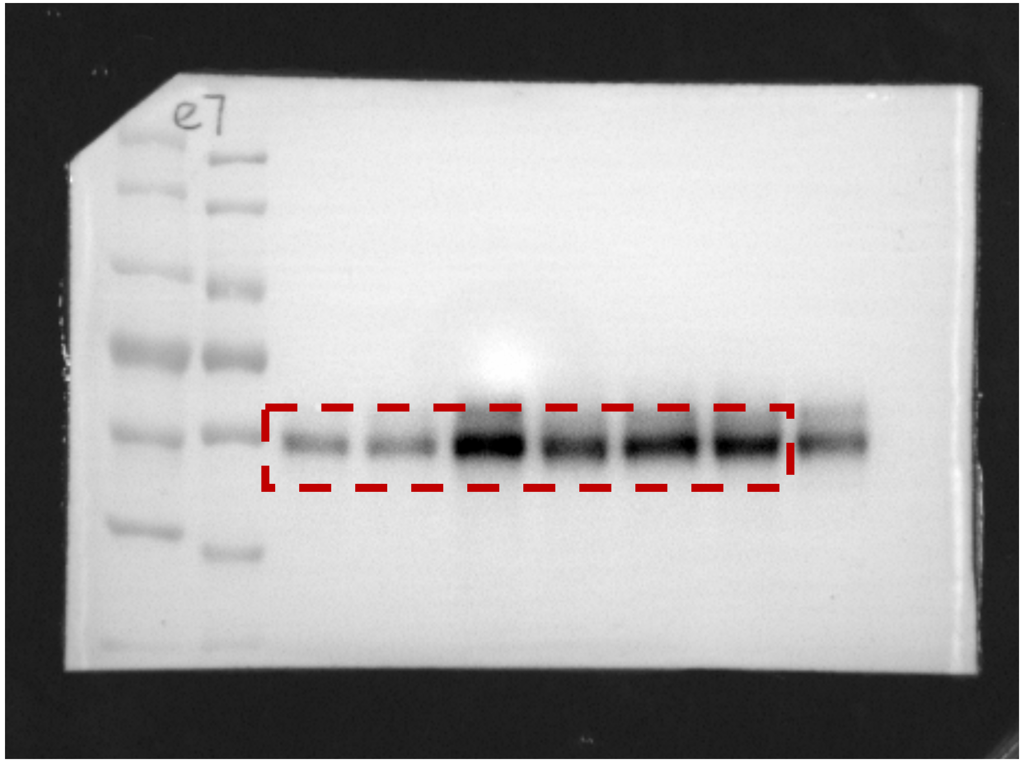

PLIN1

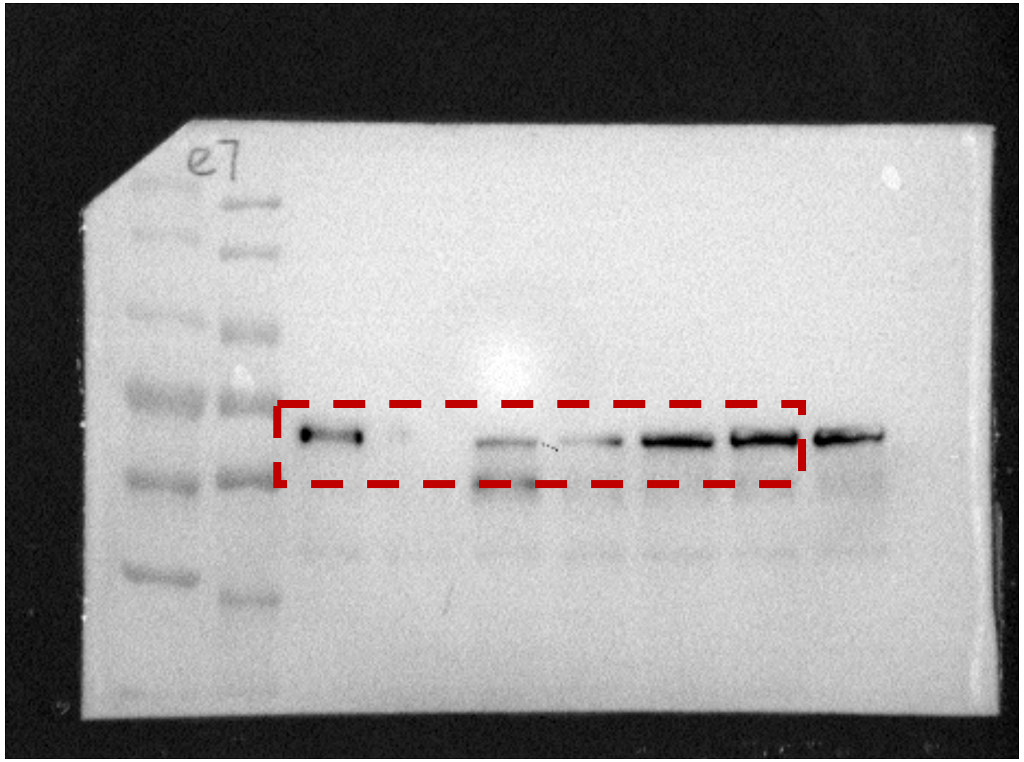

Full unedited gel for Supplemental Figure 6L

pCREB

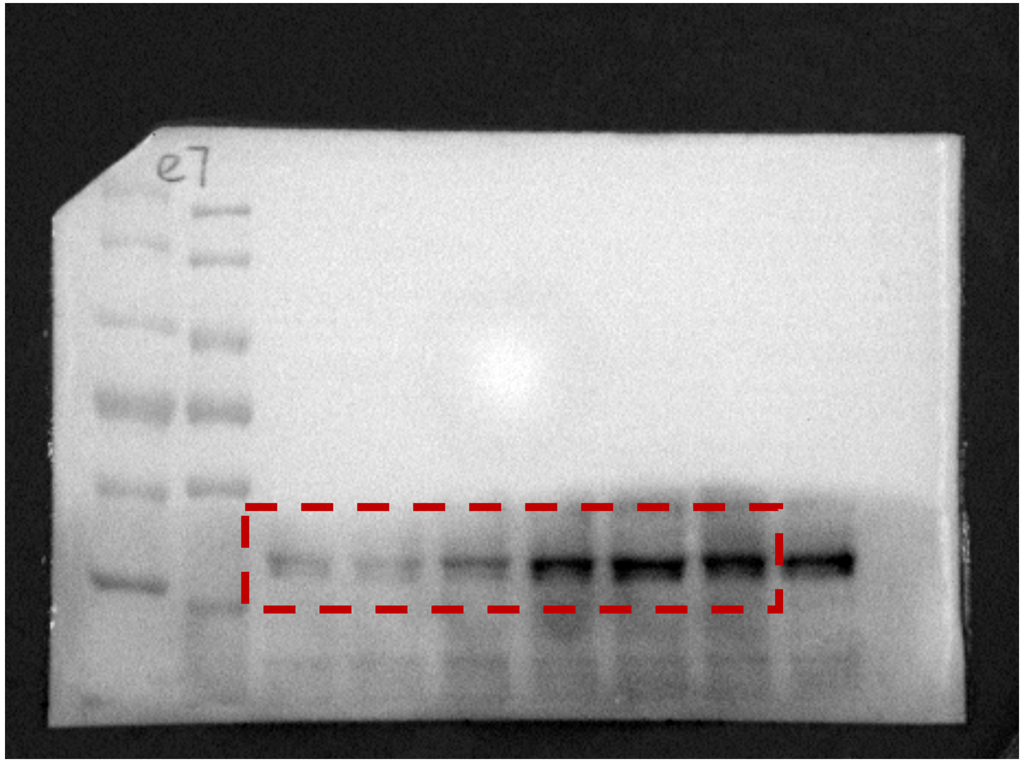

CREB

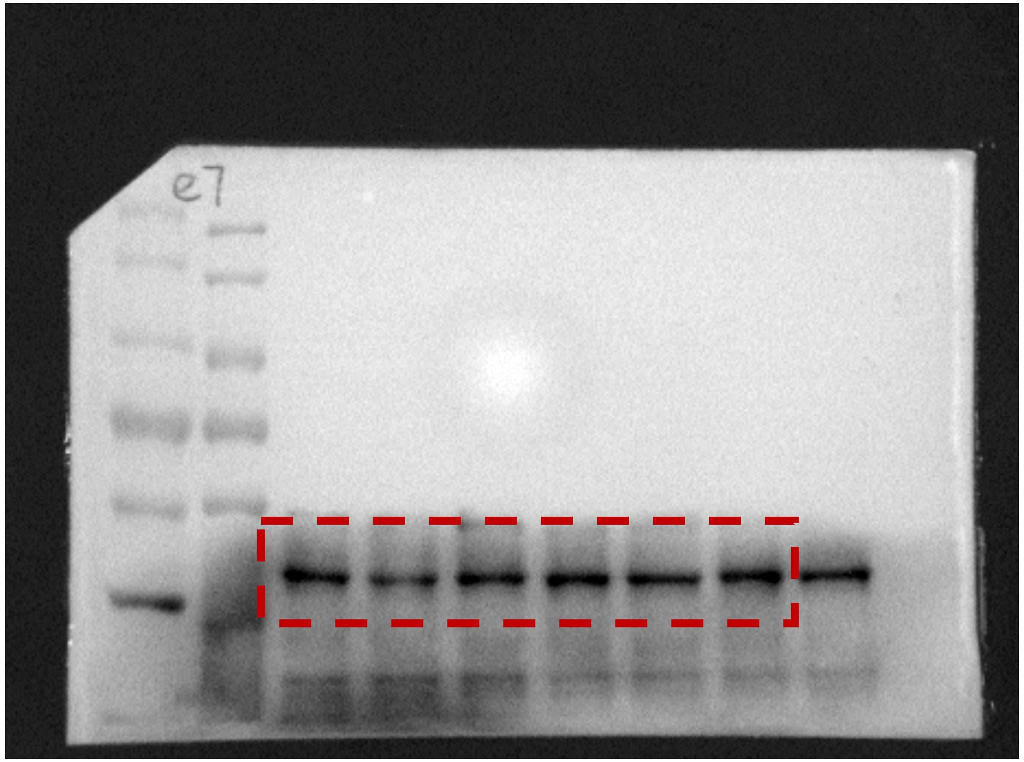

Full unedited gel for Supplemental Figure 6L

GAPDH

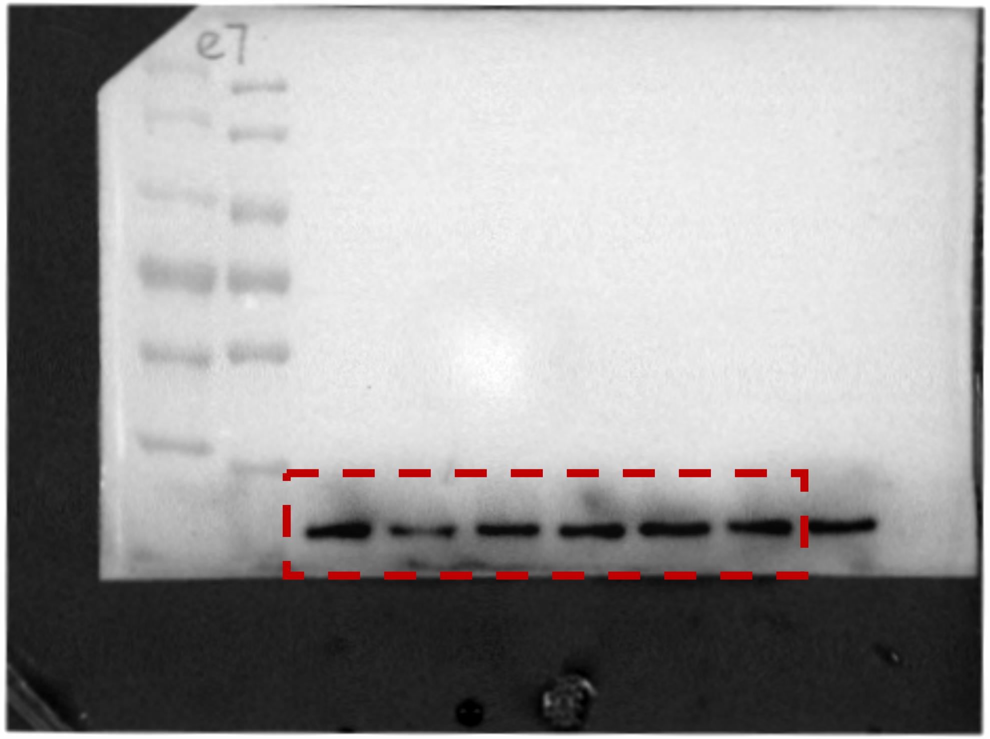

Supplement: Unedited blot and gel images [file jci-136-192355-s128.pdf]
